# A Comprehensive Comparison of Haplotype-Based Single-Step Genomic Predictions in Livestock Populations With Different Genetic Diversity Levels: A Simulation Study

**DOI:** 10.3389/fgene.2021.729867

**Published:** 2021-10-14

**Authors:** Andre C. Araujo, Paulo L. S. Carneiro, Hinayah R. Oliveira, Flavio S. Schenkel, Renata Veroneze, Daniela A. L. Lourenco, Luiz F. Brito

**Affiliations:** ^1^ Postgraduate Program in Animal Sciences, State University of Southwestern Bahia, Itapetinga, Brazil; ^2^ Department of Animal Sciences, Purdue University, West Lafayette, IN, United States; ^3^ Department of Biology, State University of Southwestern Bahia, Jequié, Brazil; ^4^ Centre for Genetic Improvement of Livestock, Department of Animal Biosciences, University of Guelph, Guelph, ON, Canada; ^5^ Department of Animal Sciences, Federal University of Viçosa, Viçosa, Brazil; ^6^ Department of Animal and Dairy Science, University of Georgia, Athens, GA, United States

**Keywords:** effective population size, genomic estimated breeding value, haplotype blocks, linkage disequilibrium, pseudo-SNP

## Abstract

The level of genetic diversity in a population is inversely proportional to the linkage disequilibrium (LD) between individual single nucleotide polymorphisms (SNPs) and quantitative trait loci (QTLs), leading to lower predictive ability of genomic breeding values (GEBVs) in high genetically diverse populations. Haplotype-based predictions could outperform individual SNP predictions by better capturing the LD between SNP and QTL. Therefore, we aimed to evaluate the accuracy and bias of individual-SNP- and haplotype-based genomic predictions under the single-step-genomic best linear unbiased prediction (ssGBLUP) approach in genetically diverse populations. We simulated purebred and composite sheep populations using literature parameters for moderate and low heritability traits. The haplotypes were created based on LD thresholds of 0.1, 0.3, and 0.6. Pseudo-SNPs from unique haplotype alleles were used to create the genomic relationship matrix ( G ) in the ssGBLUP analyses. Alternative scenarios were compared in which the pseudo-SNPs were combined with non-LD clustered SNPs, only pseudo-SNPs, or haplotypes fitted in a second G (two relationship matrices). The GEBV accuracies for the moderate heritability-trait scenarios fitting individual SNPs ranged from 0.41 to 0.55 and with haplotypes from 0.17 to 0.54 in the most (Ne ≅ 450) and less (Ne < 200) genetically diverse populations, respectively, and the bias fitting individual SNPs or haplotypes ranged between −0.14 and −0.08 and from −0.62 to −0.08, respectively. For the low heritability-trait scenarios, the GEBV accuracies fitting individual SNPs ranged from 0.24 to 0.32, and for fitting haplotypes, it ranged from 0.11 to 0.32 in the more (Ne  ≅ 250) and less (Ne  ≅ 100) genetically diverse populations, respectively, and the bias ranged between −0.36 and −0.32 and from −0.78 to −0.33 fitting individual SNPs or haplotypes, respectively. The lowest accuracies and largest biases were observed fitting only pseudo-SNPs from blocks constructed with an LD threshold of 0.3 (*p* < 0.05), whereas the best results were obtained using only SNPs or the combination of independent SNPs and pseudo-SNPs in one or two G matrices, in both heritability levels and all populations regardless of the level of genetic diversity. In summary, haplotype-based models did not improve the performance of genomic predictions in genetically diverse populations.

## 1 Introduction

Genomic selection (GS) (Meuwissen et al., 2001) is now routinely used worldwide in livestock and plant breeding programs (Lourenco et al., 2020; Moreira et al., 2020). GS enables the prediction of more accurate genomic estimated breeding values (GEBVs) at earlier stages compared to the traditional pedigree-based evaluation (Brito et al., 2017a; Guarini et al., 2018, 2019). The advantages of GS compared to the pedigree-based are even greater for lowly-heritable traits, traits measured late in life, and sex-limited or expensive-to-measure traits (Daetwyler et al., 2012; Lourenco et al., 2020).

Over the past 15–20 years, several statistical methods have been proposed aiming to obtain more accurate and less biased GEBVs. Among the available methods, the single-step genomic best linear unbiased prediction (ssGBLUP; Legarra et al., 2009; Aguilar et al., 2010) is widely used to perform genomic predictions in livestock. This method enables the simultaneous evaluation of both genotyped and non-genotyped individuals and has similar or better statistical properties and predictive ability compared to other approaches such as pedigree-based BLUP and multi-step GBLUP (Aguilar et al., 2010; Legarra et al., 2014; Guarini et al., 2018; Piccoli et al., 2020).

Although the pioneer GS study (i.e., Meuwissen et al., 2001) fitted single nucleotide polymorphism (SNP) haplotypes as covariates in the models, subsequent studies were mainly performed based on individual SNPs. This is most likely due to the additional analytic steps and higher computational requirements when fitting haplotype-based models. In this sense, it is important to first define the haplotype blocks or haploblocks, which are sizable regions of the genome with little evidence of historical recombination (Gabriel et al., 2002), i.e., a genomic region between two or more marker loci. More recently, the use of haplotypes as covariates in genomic evaluations rather than single SNPs has been further investigated due to many potential advantages. Haplotypes are more polymorphic than individual SNPs because they can be multi-allelic (Meuwissen et al., 2014) and they can be in stronger linkage disequilibrium (LD) with Quantitative Trait Loci (QTLs) compared to individual SNPs with low minor allele frequency (MAF) (Hess et al., 2017). In this context, the potential stronger LD between haplotypes and QTL in comparison to individual SNPs can yield more accurate GEBVs (Calus et al., 2008; Cuyabano et al., 2014; 2015). Moreover, haplotype alleles have the potential to capture epistatic effects within blocks and the QTL can be flanked by SNPs that delimit the haploblock (Hess et al., 2017; Jiang et al., 2018; Karimi et al., 2018).

Previous studies based on simulated data have shown that fitting haplotypes can substantially improve the performance of genomic predictions compared to individual SNP-based methods (Calus et al., 2008; Villumsen et al., 2009). However, none or only small increases in the predictive ability of GEBVs have been observed in practice (e.g., Cuyabano et al., 2014, 2015; Hess et al., 2017; Karimi et al., 2018; Mucha et al., 2019; Won et al., 2020). The large majority of the studies evaluating haplotype-based models were done in dairy cattle populations (real or simulated datasets), which usually have high LD levels between SNP markers and lower genetic diversity (Ne lower than 100; Makanjuola et al., 2020). Haplotype-based genomic predictions in populations with increased genetic diversity, on the other hand, have not been widely explored yet, and the knowledge of their possible advantages is limited (Feitosa et al., 2019; Teissier et al., 2020).

Different from intensively selected populations and pure breeds, which present low genetic diversity (e.g., Holstein dairy cattle), genetically diverse populations (e.g., relatively recent breeding programs in small ruminants and crossbred or composite populations) may have more alleles segregating in the haplotype blocks and greater complexity in the interactions among haplotype allele effects within haploblocks. Thus, we hypothesize that haplotype-based methods could result in more accurate and less biased GEBV prediction when compared to SNP-based models in populations with high genetic diversity because of their development process (e.g., relatively lower selection pressures, crossbreeding) and more complex haplotype structure than observed in populations with low genetic diversity. Simulated data is an interesting approach to investigate this hypothesis because the true breeding values (TBVs) are known (Morris et al., 2019; Oliveira et al., 2019). Therefore, we simulated sheep populations with different genetic diversity levels to test our hypothesis. Sheep is a good model due to the large genetic diversity in commercial populations, with Ne ranging from less than 50 to over 1,000 (Kijas et al., 2012; Brito et al., 2017b; Stachowicz et al., 2018). Hence, the main objective of this study was to evaluate the accuracy and bias of GEBVs in genetically diverse populations, using ssGBLUP when: 1) only individual SNPs are used to construct a single genomic relationship matrix ( G ); 2) non-clustered (out of haploblocks) SNPs and haplotypes (fitted as pseudo-SNPs) are used to construct a single G ; 3) only haplotypes are used to construct a single G ; and 4) non-clustered SNPs and haplotypes are used to construct two G matrices. We also compared the impact of different SNP panel densities and haploblock-building methods on the performance of genomic prediction, as these factors could impact the accuracies and bias of genomic predictions.

## 2 Materials and Methods

The approval of Institutional Animal Care and Use Committee was not required because this study only used computationally simulated datasets.

### 2.1 Data Simulation

#### 2.1.1 Population Structure

**FIGURE 1:**
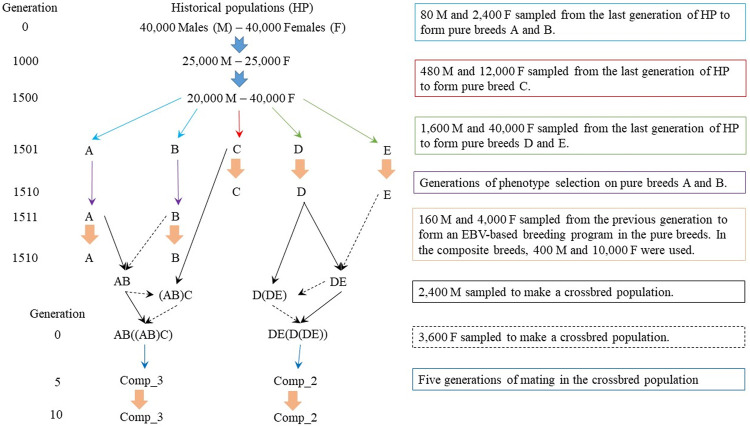
Simulation design to obtain pure and composite sheep populations.

The simulation was performed to mimic datasets of purebred and composite sheep populations (Kijas et al., 2012; Prieur et al., 2017; Brito et al., 2017a; Oliveira et al., 2020). The QMSim software (Sargolzaei and Schenkel, 2009) was used to simulate a historical population initially with 80,000 individuals (40,000 males and 40,000 females). Then, a population bottleneck was simulated, reaching 50,000 individuals (25,000 males and 25,000 females) in the 1,000th generation. After that, there was an increase in the population to 60,000 individuals, with 20,000 males and 40,000 females in the 1,500th generation. There was random mating in the historical population, with gametes randomly sampled from the pool of males and females present in each generation. Mutation and genetic drift were considered in the historical population to create the initial LD. The complete simulation design is summarized in Figure 1.

Five random samples from the last historical population were selected to create five pure breeds, called A, B, C, D, and E (Figure 1). The combination of different founder population sizes (2,480 animals for the breeds A and B, 12,480 for the breed C, and 41,600 for the breeds D and E) and generations of phenotypic selection (10 for the breeds A and B, and one generation for the breeds C, D, and E) were used to achieve different LD patterns and, consequently, different Ne in the most recent populations. There were random matings and exponential increase in the number of females in a rate of 0.10 for the breeds A and B and 0.15 for the breeds C, D, and E. During the generations of phenotypic selection, it can be considered that the breeds were separated geographically, restricting the mating within each population. Subsequently, the pure breeds were divergently selected based on estimated breeding values (EBVs) predicted using BLUP, with breeds A, C, and D selected for increasing and breeds B and E for decreasing the EBVs for the simulated trait. All breeds were selected based on the EBVs during 10 generations. The male/female ratio in the EBV-selected populations was 1/25, with a replacement rate of 40% for males and 20% for females. There were single, double, and triple births, with the odds of 30, 50, and 20%, respectively, to be similar with the ones observed in sheep flocks. The number of individuals in each generation of EBV-based selection were tested and at the end were greater than 7,000 to allow a reasonable number of selection candidates in each generation.

Crosses were made to obtain composite breeds, which had two or three pure breeds as the starting point (Figure 1). Two composite populations were created based on either two breeds (Comp_2), which had 62.5% of breed D and 37.5% of breed E (Figure 1), or three breeds (Comp_3), which had 37.5% of breed A, 37.5% of breed B, and 25.0% of breed C (Figure 1). Random mating was restricted within each crossbreed population for five generations. According to Rasali et al. (2006), five-to-six generations are sufficient to stabilize the frequencies of linked genes in new populations. Thereafter, the composite breeds were divergently selected using EBVs for the next 10 generations, with Comp_2 and Comp_3 divergently selected for decreasing and increasing performance, respectively. Mating type, sire and dam replacements, and the number of births per dam in the composite breeds were the same as those previously described for the pure breeds. The number of individuals per generation in the composite breeds (during the selection based on EBVs) was more than 18,000, to keep a higher Ne on those populations compared to the pure breeds.

#### 2.1.2 Effective Population Size in the Recent Populations

The number of generations in the pure breeds during the expansion of the recent populations were modified accordingly to achieve the LD patterns corresponding to Ne of ∼100, ∼250, and ∼500. The Ne was calculated using the LD and the realized inbreeding in the recent populations for pure and composite breeds under EBV-based selection. With the LD approach, Ne was estimated using the formula: $Ne_{LD}={\left(4c\right)}^{-1}\left\{{\left[E\left(r^{2}\right)\right]}^{-1}-2\right\}$ , which is a re-arrangement of the estimator $E\left(r^{2}\right)={\left(4Nec+2\right)}^{-1}$ proposed by Sved (1971), where $E\left(r^{2}\right)$ is the expected LD for a population with effective size Ne, c is the genetic distance (chromosome segment size in Morgans—M) within autosomal chromosomes. It was considered that 1 Mb corresponds to a centimorgan (cM) when calculating the c value, as this is an acceptable approximation in sheep (Prieur et al., 2017). Lastly, populations were simulated to have an LD of approximately 0.024, 0.010, and 0.005 for SNPs spaced apart by 10 Mb, which correspond to the values of $E\left(r^{2}\right)$ for Ne = 100, 250, and 500, respectively. A 10 Mb distance corresponds to an Ne that existed five generations ago (considered as current Ne), based on the relationship t=1/2c proposed by Hayes et al. (2003), where t is the number of generations ago and c is as previously defined. Estimation of LD was performed considering only SNPs with MAF higher than 0.05 using the $r^{2}\;$ metric (Hill and Robertson, 1968). We also estimated the Ne based on the realized inbreeding five generations ago using the formula (Falconer and Mackay, 1996): $Ne_{Inb}=1/2\Delta F$ , where $\Delta F=\left(F_{n}-F_{n-1}\right)/\left(1-F_{n-1}\right)$ and $F_{n}$ is the average inbreeding in the *n*th generation. The average inbreeding per generation was obtained from the QMSim software outputs (Sargolzaei and Schenkel, 2009).

#### 2.1.3 Simulated Traits

We simulated two traits with initial heritability levels of 0.30 and 0.10 (global parameters for the QMSim software; Sargolzaei and Schenkel, 2009), to represent moderate (MH2) and low (LH2) additive genetic effects, respectively, affecting the total phenotypic variability of the trait. The phenotypic variance was set to 100 in both simulations. The heritability was estimated in the recent populations based on pedigree and phenotype information using the AIREMLf90 software (Misztal et al., 2018) to verify if the desired values were achieved. All simulations were replicated five times using different seed values in order to simulate different populations. Only additive genetic effects were simulated due to the QMSim software (Sargolzaei and Schenkel, 2009) capabilities.

#### 2.1.4 Genome and Data Editing

The genome was simulated with 26 autosomal chromosomes with size varying between 43 and 301 cM (a total of 2,656 cM), mimicking the sheep genome (Supplementary Material S1). The number and size of chromosomes were defined based on information obtained from the most recent sheep reference genome (assembly OAR_v4.0) available in the NCBI platform (www.ncbi.nlm.nih.gov/genome?term=ovis%20aries). The genome simulation was also performed using the QMSim software (Sargolzaei and Schenkel, 2009).

A total of 3,057 QTLs were simulated, spanning the whole autosomal genome. The number of QTLs per chromosome varied between 51 and 391 (Supplementary Material S1), which was chosen based on the information published in the AnimalQTLdb (AnimalQTLdb, 2019). QTLs with the number of alleles varying from two to six were simulated to evaluate the advantages of using haplotype-based approaches. All simulated markers were bi-allelic to mimic SNP markers, and the total number of SNPs was set to 576,595 (Supplementary Material S1; similar number of autosomal SNPs included in the Ovine Infinium® HD SNP Beadchip 600K; FarmIQ, 2013; Kijas et al., 2014) sampled from the segregating loci (MAF ≥0.05) in the last historical generation. The information on the number of markers in each chromosome was obtained from the SNPchiMp v.3 platform (Nicolazzi et al., 2015). Both QTL and markers were randomly distributed within chromosome and placed in different chromosomic positions, i.e., simulated QTLs were not among the SNPs, so that the genomic predictions rely only on the LD between them.

The additive genetic effects of the QTL were sampled from a gamma distribution with the shape parameter equal to 0.4, whereas no effects were simulated for the SNP markers. The initial allele frequencies assumed for QTL and markers (generation 0 of the historical population) were 0.5. The QTL heritability on the MH2 and LH2 traits was equal to 50 and 10% of the trait heritability, i.e., 0.15 and 0.01, respectively. The remaining genetic variance not explained by the QTLs was attributed to the polygenic effect. Recurrent mutation rates on the order of 1 × 10^−4^ were simulated for the QTL and markers. Rates of 0.05 and 0.01 were used for the occurrence of missing genotypes and genotyping errors, respectively.

Quality control (QC) was performed in the genotype file of each simulated recent population for each replicate, using the PREGSf90 software from the BLUPf90 family programs (Misztal et al., 2018). In this step, SNPs with no extreme departure from Hardy–Weinberg equilibrium (difference between observed and expected frequency of heterozygous less than 0.15) and MAF ≥0.01 were maintained. All SNPs passed this QC for all populations, indicating that there was enough variability on the simulated SNP chip panel.

### 2.2 Haplotype Blocks Construction

The FImpute v.3.0 software (Sargolzaei et al., 2014) was used to phase the genotypes (i.e., to infer SNP allele inheritance). Subsequently, the haploblocks were constructed using different LD thresholds (variable haploblock sizes), as described below. The $r^{2}$ metric (Hill and Robertson, 1968) was used to calculate the LD between markers to construct the haploblocks, as this measure is less sensitive to allele frequency (Bohmanova et al., 2010). The “gpart” package (Kim et al., 2019) implemented in the R software (R Core Team, 2020) was used to build the haploblocks considering $r^{2}$ levels of 0.1 (low), 0.3 (moderate), and 0.6 (high) based on the Big-LD approach (Kim et al., 2018). Following the previous definition of haploblocks (Gabriel et al., 2002), a haploblock in this study was considered as a genomic region spanning at least two SNPs.

### 2.3 Prediction of GEBV

All genomic predictions were performed using the ssGBLUP method implemented in the BLUPf90 family programs (Misztal et al., 2018). Before using the BLUPf90 software, the AIREMLf90 software (Misztal et al., 2018) was used to estimate the variance components for each simulation replicate for the models described in the next sections.

#### 2.3.1 ssGBLUP Using SNPs

The model used to predict the GEBVs under this approach was

y=Xb+Zu+e

where y is an N × 1 vector of phenotypes for genotyped and non-genotyped animals, b is the vector of fixed effects (i.e., generation), u is a random vector of GEBVs for genotyped and non-genotyped animals with $u\;∼N\left(0,H\sigma_{g}^{2}\right)$ , e is the vector of random errors with $e\;∼N\left(0,I\sigma_{e}^{2}\right)$ , X is the incidence matrix of fixed effects, and Z is the incidence matrix that relates the records to GEBVs. In the case of ssGBLUP fitting individual SNPs, the H matrix is a hybrid relationship matrix that combines the genomic and pedigree relationships (Legarra et al., 2009), and its inverse can be computed directly in the mixed model equations as follows (Aguilar et al., 2010):

$$H^{-1}=A^{-1}+\left[\begin{array}{cc} 0 & 0\\ 0 & \tau {\left(\alpha G+\beta A_{22}\right)}^{-1}-\omega A_{\mathrm{22}}^{-1} \end{array}\right]$$

where $A^{-1}$ is the inverse of pedigree relationship matrix, $A_{22}^{-1}$ is the inverse of pedigree relationship matrix for the genotyped animals, and G is the genomic relationship matrix. The G matrix was constructed as in the first method proposed by Vanraden (2008):

$$\mathrm{G=}\frac{\mathrm{MM\prime}}{2\Sigma p_{i}\left(1-p_{i}\right)}$$

where M is the matrix of centered genotypes, with a dimension equal to the number of animals by the number of markers. The blending and weighting parameters for the genomic information were the default values in the PREGSf90 software ( α and β equal to 0.95 and 0.05, respectively, and τ and ω equal to 1.0; Misztal et al., 2018).

#### 2.3.2 ssGBLUP Using SNPs and Haplotypes Combined in a Single Genomic Relationship Matrix

The model and assumptions in this approach are the same as described in *ssGBLUP using SNPs*. However, the G matrix used to construct the combined relationship in this model had both independent markers (i.e., non-blocked markers, which are SNPs out of the LD blocks) and haplotypes as pseudo-SNPs. To build the G matrix using haplotype information, the haplotype alleles were first converted to pseudo-SNPs, as in Teissier et al. (2020). Using this approach, if there were five unique haplotype alleles in a haploblock, five pseudo-SNPs were created for this haploblock. At the end, the number of copies of a specific pseudo-SNP allele were counted and coded as 0, 1, or 2 for each individual, similar to the codes used in M (when creating the G ) as previously described based on individual SNPs. The pseudo-SNPs were subjected to the same QC steps as described above for individual SNPs.

#### 2.3.3 ssGBLUP Using Haplotypes

The model and assumptions in this approach were the same as described in *ssGBLUP using SNPs*. However, only haplotypes converted to pseudo-SNPs were used to create the G matrix used in the predictions, therefore, excluding non-blocked individual SNPs.

#### 2.3.4 ssGBLUP Using SNPs and Haplotypes Assigned to Two Different Genomic Relationship Matrices

The model used for these analyses was:

$$y=\mathrm{Xb}+Zu_{1}+\;Zu_{2}+e$$

where $u_{1}$ and $u_{2}$ are the random additive genetic effects of the first and second component of the overall GEBV, respectively, which, under this modeling, is equal to $u_{1}+u_{2}$ . All other vectors and matrices on this model are the same as described on the previous sections. The main assumption on this model is that the breeding value is divided into two uncorrelated components with their own covariance structure, being $u_{1}∼N\left(0,H_{1}\sigma_{g1}^{2}\right)$ and $u_{2}∼N\left(0,H_{2}\sigma_{g2}^{2}\right)$ , in which $H_{1}$ and $H_{2}$ are the hybrid relationship matrices with the same structure of the H matrix described before. The only difference between $H_{1}$ and $H_{2}$ is the G matrix that is combined with the pedigree relationship in each one of them, named as $G_{1}$ and $G_{2}$ , respectively, containing the genomic relationships between the individuals based on single non-blocked SNPs and haplotypes, respectively. This parametrization was used to account for the fact that haplotypes and, therefore, the corresponding pseudo-SNPs, are more polymorphic than individual SNPs. Consequently, pseudo-SNPs could better capture the effect of large-sized QTL with lower allele frequency than individual SNPs and could have different distribution of their allele effects compared to individual SNPs.

### 2.4 Training and Validation Population Sets

The populations used in the genomic predictions were the pure breeds B, C, and E, defined as Breed_B, Breed_C, and Breed_E, respectively, and composite breeds Comp_2 and Comp_3. Only breeds Breed_B, Breed_C, and Breed_E were presented here because the genetic background simulated, i.e., the size of the founder population and generations of selection, was more divergent for these populations (Figure 1). As breeds A and D had similar sizes of the founder populations and generations of selection when compared to breeds B and E, respectively, we observed similar results between breeds A and B and also D and E (data not shown).

The datasets (populations from the simulated EBV-based selection programs) were divided into training and validation sets to test the accuracy and bias of GEBVs. The training sets within each population were composed of 60,000 individuals with phenotypes randomly sampled from generations one to eight, and 8,000 of them also had genotypes for the simulated HD panel. The genotyped individuals in the training set were randomly sampled from generations four to seven. The validation populations were composed of 2,000 individuals randomly sampled from generations nine and ten and were also genotyped for the same panel. Generation eight was considered as a gap between training and validation populations in terms of genotypes. The whole pedigree (generations 1–10) was used in all analyses. As we assume that validation individuals would not have phenotypes, their GEBVs were estimated based on the relationships of the validation cohort with the training set (with phenotypes and genotypes included in the analyses).

### 2.5 Evaluated Scenarios

Although the HD SNP panel datasets were first simulated, the main genomic predictions were performed using a medium density 50 K SNP panel, which was designed based on randomly selected SNPs from the original HD panel. This step was performed because similar accuracies tend to be achieved when using a medium density SNP panel in sheep (Moghaddar et al., 2017), as well as in other species (Binsbergen et al., 2015; Ni et al., 2017; Frischknecht et al., 2018). The total number of SNPs selected for the 50 K panel was 46,827, as currently available in the 50 K SNP panel (for autosomal chromosomes) reported in the SNPchiMp v.3 platform (Nicolazzi et al., 2015). The markers in the 50 K SNP panel were randomly sampled within each autosome, and the number of SNPs per chromosome is reported in Supplementary Material S1. In addition, previous analyses showed that both SNP and haplotype-based predictions based on the HD and 50 K SNP panels were not statistically different (data not shown). Therefore, the haplotype blocks for all the prediction scenarios were created based on the 50 K panel and the results for the HD SNP panel were presented as an additional scenario.

**FIGURE 2:**
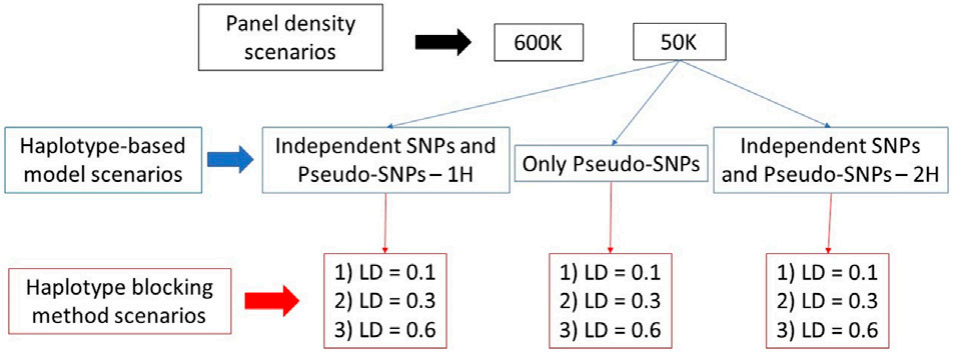
Evaluated scenarios used in the genomic predictions with pseudo-single nucleotide polymorphisms (SNPs) from linkage disequilibrium (LD) blocks using independent and pseudo-SNPs in a single genomic relationship matrix (1H), and only pseudo-SNPs and independent and pseudo SNPs in two genomic relationship matrices (2H).

At the end, 11 scenarios were evaluated, which consisted of genomic predictions using: 1) SNPs from the 600 K; 2) SNPs from the 50 K; 3–5) independent SNPs and pseudo-SNPs from haplotype blocks with LD equal to 0.1, 0.3, and 0.6 in a single relationship matrix (IPS_LD01, IPS_LD03, and IPS_LD06, respectively); 6–8) only pseudo-SNPs from haplotype blocks with LD equal to 0.1, 0.3, and 0.6 (PS_LD01, PS_LD03, and PS_LD06, respectively); and 9–11) independent SNPs and pseudo-SNPs from haplotype blocks with LD equal to 0.1, 0.3, and 0.6 in two different relationship matrices (IPS_2H_LD01, IPS_2H_LD03, and IPS_2H_LD06, respectively). All these scenarios were evaluated for two different heritability levels (moderate and low) and in each one of the five populations previously described (purebred and composite breeds with distinct Ne). Therefore, 110 different scenarios were evaluated in each one of the five replicates. A summary of the evaluated scenarios is shown in Figure 2.

### 2.6 Scenario Comparisons

The statistics related to haplotype blocking strategies were compared between populations (pure and composite breeds) within each LD threshold to create the blocks (0.1, 0.3, and 0.6), and also, the LD thresholds were compared within each population to differentiate the haplotype block structures. These statistics are: average number of haploblocks, blocked SNPs, pseudo-SNPs before and after QC, non-blocked plus pseudo-SNPs after QC, and the additional computer time required by using pseudo-SNPs (e.g., SNPs phasing, haplotype blocking, and pseudo-SNP derivation). The GEBV accuracies and bias in each prediction scenario were compared within each population, to mimic population-specific (breed) genetic evaluation. Prediction accuracy was estimated as the Pearson correlation coefficient between the GEBVs and TBVs for the validation animals, for each replicate and scenario. Prediction bias was assessed as the deviation from one of the linear regression coefficients ( $\beta_{1}$ ) of the TBVs on the GEBVs ( $i.e.,\;bias=\;\beta_{1}-1;\mathrm{where}\;TBV=\beta_{0}+\beta_{1}\times GEBV$ ) in the validation population in each replicate and scenario.

A linear mixed model was used to test the effect of the population and LD level on the statistics from haplotype block strategies and the effect of marker information (SNP and haplotype prediction scenarios) on the accuracy and bias of GEBV prediction. The statistical model used was:

$$y_{ij}=\mu +T_{i}+R_{j}+\varepsilon_{ij}$$

where $/y_{ij}$ is the observation of the *i*th treatment on the *j*th repetition; $T_{i}$ is the treatment effect, in which *i* is equal to Breed_B, Breed_C, Breed_E, Comp_2, and Comp_3 to compare the population effect over the statistics from haplotype block strategies within each LD threshold; equal to LD01, LD03, and LD06 to compare the effects of LD level over the statistics from haplotype block strategies within population; and equal to 600 K, 50 K, IPS_LD01, IPS_LD03, IPS_LD06, PS_LD01, PS_LD03, PS_LD06, IPS_2H_LD01, IPS_2H_LD03, and IPS_2H_LD06 to test the effect of marker information over the accuracy and bias of GEBV prediction within each population; $R_{j}$ is the random effect of replicates which was assumed to follow $∼N\left(0,B\sigma_{b}^{2}\right)$ ; and $\varepsilon_{ij}$ is the residual effect of the model.

Replicate was used as a random effect in the model to account for the covariance between the scenarios, as the compared averages were obtained within the simulated populations in each replicate. This was done to reduce the occurrence of false negatives (Type-II error). Different covariance structures ( B ) were evaluated (spherical, compound symmetry, simple autoregressive process, and unstructured covariance) to explain the covariances between replicates, and the structure that presented the lowest Akaike information criterion (AIC) and Bayesian information criterion (BIC) values was used in the final models for comparison purposes. After defining the appropriate covariance structure (which was not the same for all scenarios, with unstructured covariance being the best in the major part of the scenarios), the means of the $T_{i}$ levels were compared using the Tukey test at 5% of significance level. The “nlme” (Pinheiro et al., 2021) and “emmeans” (Lenth, 2021) R packages were used to fit the models and compare the means, respectively, in the R environment (R Core Team, 2020).

## 3 Results

### 3.1 Genetic Diversity and Genetic Parameters in the Simulated Populations

After the simulation process, several different Ne levels were observed in the recent populations studied (generations 1–10 of pure and composite breeds under EBV-based selection). The total additive genetic effect variances estimated with the models that used two H matrices (*ssGBLUP using SNPs and Haplotypes Assigned to Two Different Genomic Relationship Matrices*), taken as $\sigma_{g1}^{2}+\sigma_{g2}^{2}$ , and the residual variances were similar to the variances estimated with the models that fitted a single H matrix (*ssGBLUP using SNPs*, *ssGBLUP using SNPs and Haplotypes Combined in a Single Genomic Relationship Matrix*, *ssGBLUP Using Haplotypes*) and similar to the variances estimated with the model that used only the pedigree relationship matrix (*Simulated Traits*; Supplementary Materials S3, S4). Therefore, for simplicity, only the genetic parameters estimated based on the pedigree relationship matrix are presented in Table 1. A population structure analysis based on principal components (PCs) of the G matrix using the SNPs from the 50 K panel was also performed (Supplementary Material S2). Individuals within the population were close to each other, and no clear clusters between populations existed at 95% confidence level based in the approximated unbiased test from a hierarchical clustering method using 10,000 bootstrap samples (Shimodaira, 2002; Supplementary Material S2).

**TABLE 1:** Average (SE) effective population size based on the linkage disequilibrium (Ne_LD_) and realized inbreeding (Ne_Inb_) methods, additive genetic variance ( $\sigma_{a}^{2}$ ), residual variance ( $\sigma_{a}^{2}$ ), and heritability (h^2^) estimates of the trait in simulated sheep populations.

Simulation	Population^a^	Ne_LD_ ^b^	Ne_Inb_ ^c^	$\sigma_{a}^{2}$	$\sigma_{e}^{2}$	H^2^
Moderate h^2^ (0.30)	Breed_B	110 (6)	190 (17)	27.12 (0.27)	71.54 (0.10)	0.27 (0.00)
	Breed_C	379 (8)	260 (15)	28.09 (0.25)	70.85 (0.26)	0.29 (0.00)
	Breed_E	359 (5)	192 (6)	27.45 (0.35)	72.42 (0.34)	0.28 (0.00)
	Comp_2	644 (15)	446 (7)	25.82 (0.37)	73.07 (0.25)	0.26 (0.00)
	Comp_3	466 (40)	447 (53)	26.80 (0.62)	72.88 (0.50)	0.27 (0.00)
Moderate h^2^ (0.10)	Breed_B	125 (8)	94 (11)	9.17 (0.26)	90.30 (0.38)	0.09 (0.00)
	Breed_C	272 (11)	120 (11)	9.31 (0.28)	89.91 (0.23)	0.09 (0.00)
	Breed_E	251 (22)	119 (19)	9.31 (0.23)	90.38 (0.26)	0.09 (0.00)
	Comp_2	522 (32)	259 (40)	8.42 (0.27)	91.13 (0.27)	0.08 (0.00)
	Comp_3	407 (32)	235 (38)	8.00 (0.29)	91.90 (0.23)	0.08 (0.00)

a Breed_B, Breed_C, and Breed_E: simulated pure breeds with different genetic backgrounds; Comp_2 and Comp_3: composite breeds based on two and three pure breeds, respectively.

b Estimated based on the re-arranged estimator present in Sved (1971).

c Estimated based on the formula presented by Falconer and Mackay (1996).

#### 3.1.1 Ne and Genetic Parameters for the Simulation of a Trait With Moderate Heritability

The average Ne_LD_ ranged between 110 and 644 (Breed_B and Comp_2, respectively), while the Ne_Inb_ varied from 159 to 373 (Breed_B and composite breeds, respectively), being lower in pure breeds independently of the Ne measure (Table 1 and Supplementary Material S3). The average additive genetic variance in the MH2 scenarios ranged from 25.82 (Comp_2) to 28.09 (Breed_C), while the residual variances ranged from 70.85 (Breed_C) to 73.07 (Comp_2). Average heritability estimates ranging from 0.26 (Comp_2) to 0.29 (Breed_C) were observed across populations, which are close to the global simulation parameters (heritability and phenotypic variance equal to 0.30 and 100, respectively).

#### 3.1.2 Ne and Genetic Parameters for the Simulation of a Low Heritability Trait

The average Ne_LD_ ranged from 125 (Breed_B) to 522 (Comp_2), while Ne_Inb_ ranged between 94 and 259 for these same populations (Table 1 and Supplementary Material S4). Average additive genetic variances ranging from 8.00 (Comp_3) to 9.31 (Breed_C and Breed_E) were observed. The average residual variances ranged from 90.30 (Breed_B) to 91.90 (Comp_3). In the LH2 scenarios, the average heritabilities were equal to 0.09 in the pure breeds and 0.08 in the composite breeds, which are close to the global simulation parameters (heritability and phenotypic variance equal to 0.10 and 100, respectively).

### 3.2 Statistics From Haplotype Blocks and Pseudo-SNPs: Moderate Heritability Trait

#### 3.2.1 Number of Blocks

**FIGURE 3:**
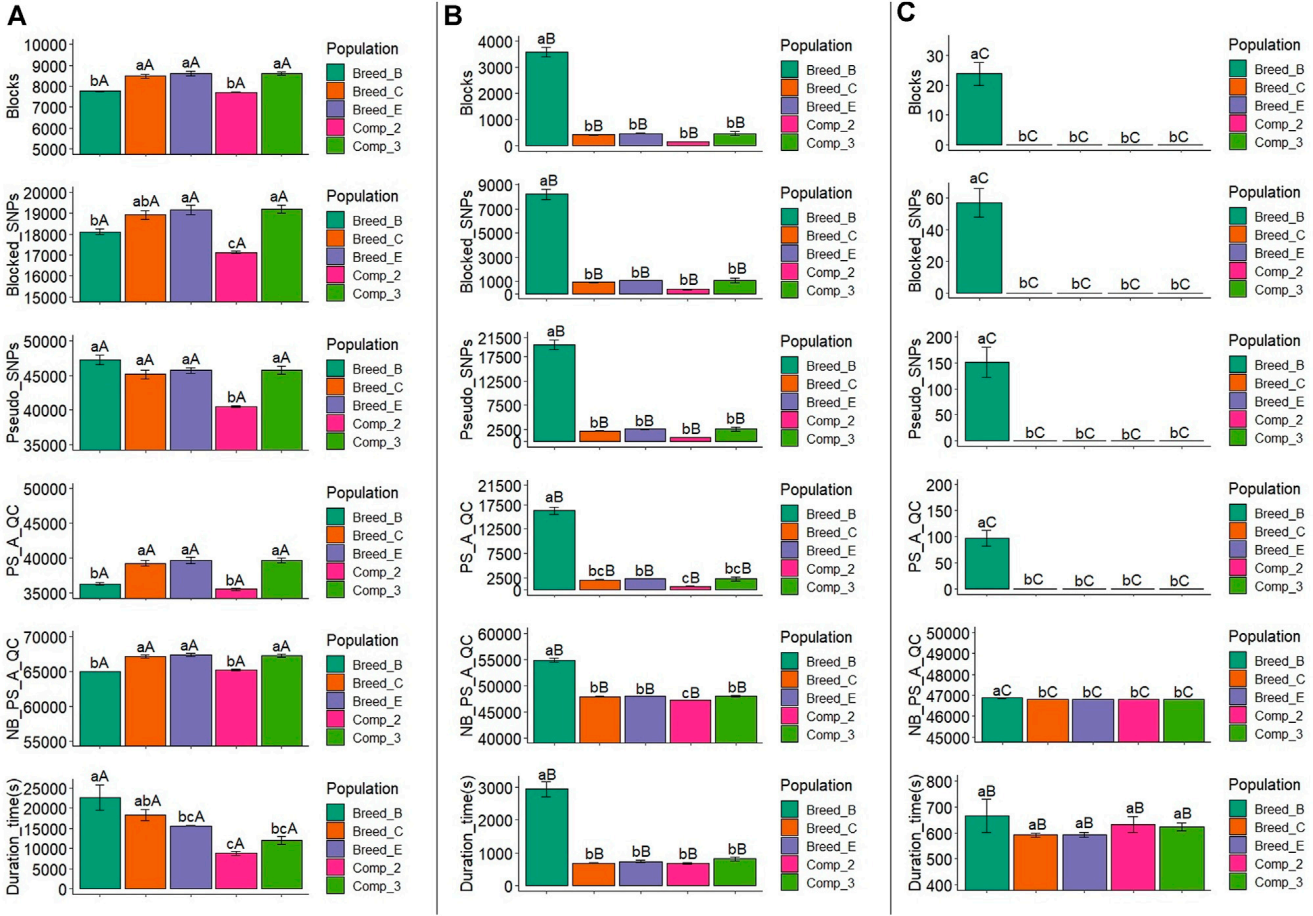
Average number of blocks (Blocks) spanning two or more SNPs, markers within blocks (Blocked_SNPs), pseudo-SNPs (Pseudo_SNPs), pseudo-SNPs after quality control (PS_A_QC), non-blocked SNPs plus pseudo-SNPs after quality control (NB_PS_A_QC), and computing time to obtain the pseudo-SNPs (Duration_time) in the simulation for a trait with moderate heritability (h^2^ = 0.30). A, B, and C show the results for haplotype blocks with LD thresholds of 0.1, 0.3, and 0.6, respectively. Breed_B, Breed_C, and Breed_E: simulated pure breeds with different genetic backgrounds; Comp_2 and Comp_3: composite breeds from two and three pure breeds, respectively. The same lower- or upper-case letters mean no statistical difference comparing populations within LD thresholds and LD threshold across populations, respectively, at 5% significance level by the Tukey test.

The average number of blocks with two or more SNPs and the LD threshold equal to 0.1 ranged from 7,709.6 (Comp_2) to 8,607.6 (Comp_3), with Comp_2 and Breed_B showing similar and significantly lower number of blocks with this LD threshold level than the other populations (Figure 3A and Supplementary Material S5). With the LD threshold equal to 0.3, the average number of blocks ranged from 145.0 (Comp_2) to 3,574.6 (Breed_B), and Breed_B showed significantly larger mean compared to the other populations (Figure 3B and Supplementary Material S5). Only Breed_B had blocks with an LD threshold equal to 0.6, with an average equal to 23.8, which was statistically different from all the other populations (Figure 3C and Supplementary Material S5). Within each population, the mean number of blocks from LD threshold levels of 0.1, 0.3, and 0.6 were statistically different for all populations, with the LD threshold equal to 0.1 being the largest, followed by the LD threshold equal to 0.3, and the 0.6 level yielding the lowest number of blocks.

#### 3.2.2 Number of Blocked SNPs

The average number of blocked SNPs for the LD threshold equal to 0.1 varied between 17,122.2 (Comp_2) and 19,199.8 (Comp_3) (Figure 3A and Supplementary Material S5), and for Comp_2, it was significantly lower than all the other populations. The average number of SNPs within blocks with an LD threshold equal to 0.3 ranged from 340.4 (Comp_2) to 8,195.4 (Breed_B) (Figure 3B and Supplementary Material S5). The number of blocked SNPs for Breed_B was significantly higher than for the other populations (which did not differ among them). The average number of blocked SNPs with LD threshold equal to 0.6 in Breed_B was 56.8 (Figure 3C and Supplementary Material S5) and was significantly greater, as no blocks were created for all the other populations.

#### 3.2.3 Number of Pseudo-SNPs After Quality Control

After QC, the average number of pseudo-SNPs from blocks with an LD threshold equal to 0.1 was reduced, ranging from 35,524.6 (Comp_2) to 39,713 (Breed_E) (Figure 3A and Supplementary Material S5). In general, Breed_B and Comp_2 were statistically similar and had lower averages compared to all other populations. The average number of pseudo-SNPs after QC with haploblocks constructed with the LD threshold of 0.3 was between 718.6 (Comp_2) and 16,259.4 (Breed_B), in which only Breed_B was statistically different from all other populations (Figure 3B and Supplementary Material S5). With an LD threshold equal to 0.6, the average number of pseudo-SNPs for Breed_B was 91 and no pseudo-SNPs were generated with this LD threshold for all the other populations (Figure 3C and Supplementary Material S5). The average number of pseudo-SNPs before QC is also shown in Figure 3A and Supplementary Material S5.

#### 3.2.4 Number of Non-blocked SNPs Plus Pseudo-SNPs After Quality Control

The average number of non-blocked plus pseudo-SNPs after QC varied from 64,987.0 (Breed_B) to 67,367.2 (Breed_E) when using blocks with an LD threshold of 0.1 (Figure 3A and Supplementary Material S5). Breed_B and Comp_2 showed lower averages compared to all the other populations. Regarding the LD threshold of 0.3, the number of non-blocked plus pseudo-SNPs after QC ranged from 47,205.2 (Comp_2) to 54,891.0 (Breed_B) (Figure 3B and Supplementary Material S5). For this LD threshold, the Breed_B average was statistically greater than all the other populations. The average number of non-blocked plus pseudo-SNPs after QC was equal to 46,867.8 for Breed_B and 46,827 for all the other populations when using an LD threshold of 0.6 to create the haploblocks (Figure 3C and Supplementary Material S5).

#### 3.2.5 Additional Time to Create Pseudo-SNPs

The average computing time to create the pseudo-SNPs (also considering the haplotype phasing and blocking) was between 8,800.6 s (2 h and 26 min; Comp_2) and 22,650.0 s (6 h and 18 min; Breed_B) with the LD threshold of 0.1 (Figure 3A and Supplementary Material S5). For this LD threshold, the computing time for Breed_B was statistically similar to that in Breed_C, but significantly different from all the other populations. When using an LD threshold of 0.3 to create the blocks, the average computing time ranged from 675.4 s (11 min; Comp_2) to 2,935.0 s (49 min; Breed_B) (Figure 3B and Supplementary Material S5). The computing time for Breed_B was statistically higher than all the other populations, which were not statistically different among them. The average computing time for pseudo-SNPs from blocks with an LD threshold equal to 0.6 ranged from 591.4 (10 min) to 666.8 s (11 min) (Breed_C and Breed_B, respectively; Figure 3C and Supplementary Material S5), and no statistical differences were observed across populations. The computing time compared across LD thresholds within the population showed that LD thresholds of 0.3 and 0.6 were statistically similar and lower than with the LD threshold of 0.1.

### 3.3 Statistics From Haplotype Blocks and Pseudo SNPs: Low Heritability Trait

**FIGURE 4:**
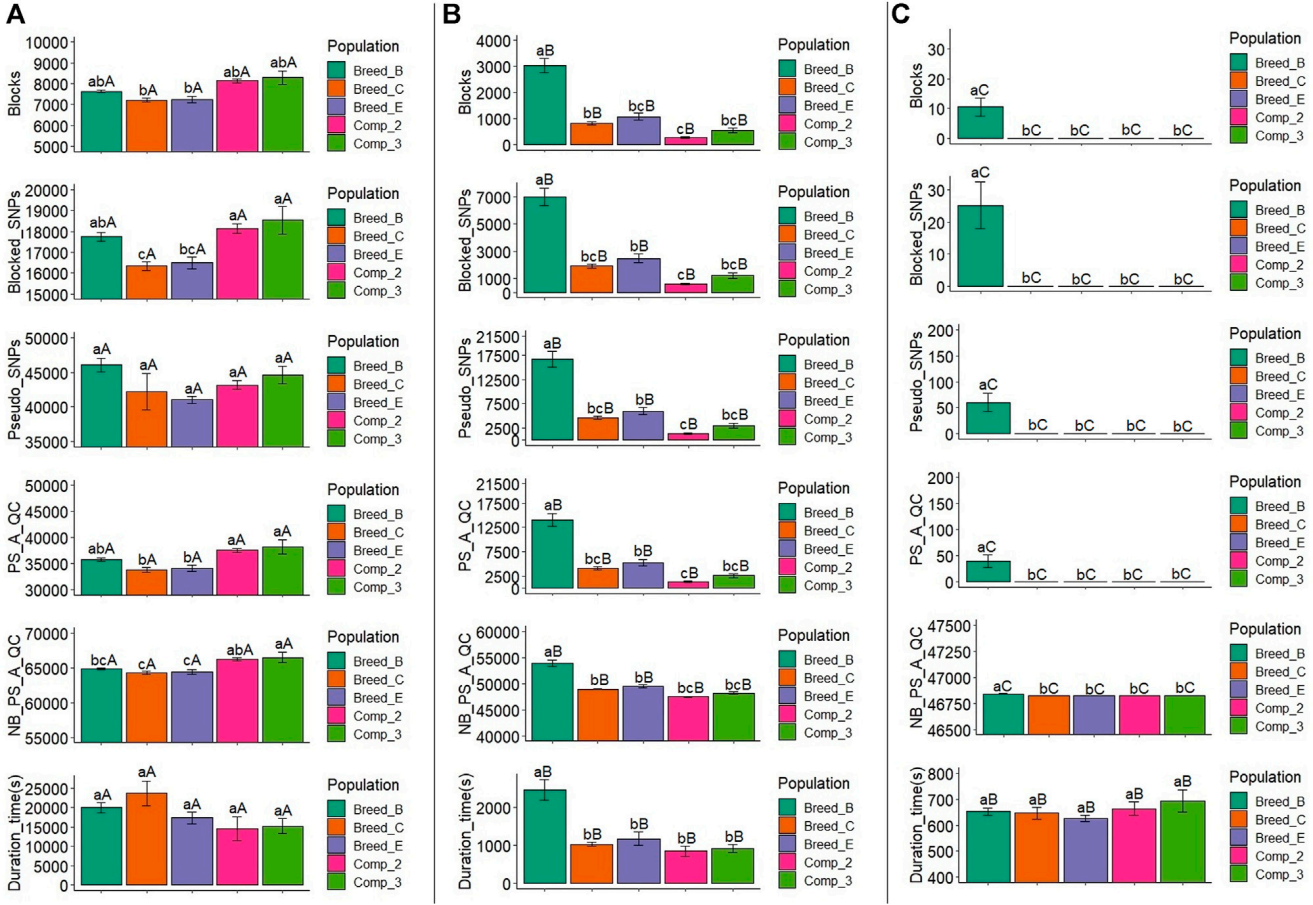
Average number of blocks (Blocks) spanning two or more SNPs, markers within blocks (Blocked_SNPs), pseudo-SNPs (Pseudo_SNPs), pseudo-SNPs after quality control (PS_A_QC), non-blocked SNPs plus pseudo-SNPs after quality control (NB_PS_A_QC), and computing time to obtain the pseudo-SNPs (Comp_time) in the simulation for a trait with low heritability (h^2^ = 0.10). A, B, and C show the results for the haplotype blocks with LD thresholds of 0.1, 0.3, and 0.6, respectively. Breed_B, Breed_C, and Breed_E: simulated pure breeds with different genetic backgrounds; Comp_2 and Comp_3: composite breeds from two and three pure breeds, respectively. The same lower- or upper-case letters mean no statistical difference comparing populations within LD thresholds and LD threshold across populations, respectively, at 5% significance level based on the Tukey test.

We have also checked the statistics from haplotype blocks and pseudo-SNPs in the low heritability trait scenarios because the simulation was done for each heritability level at a time. In general, the number of blocks, blocked SNPs, pseudo-SNPs before and after the QC, the number of non-blocked plus pseudo-SNPs after QC, and computing time to generate the pseudo-SNPs for a trait with a low heritability were similar to those for a trait with moderate heritability and are shown in Figure 4 and Supplementary Material S6. The results for the statistical comparisons in each one of these metrics for both populations, within each LD threshold, and for LD thresholds across populations were also similar between the LH2 and MH2 scenarios. The exceptions for the statistical comparisons under LH2 scenario was that the number of blocks in Breed_C and Breed_E would show a similar or lower average number of blocks, blocked SNPs, pseudo-SNPs after QC, and number of non-blocked plus pseudo-SNPs after QC than Breed_B, whereas the opposite would occur under the MH2 scenario. However, as pointed out before, the values were similar across the LH2 and MH2 scenarios. Therefore, the interpretation of the statistical comparisons for haplotype blocks in the MH2 scenario are also extended to LH2.

### 3.4 Accuracy and Bias of Genomic Predictions: Moderate Heritability Trait

#### 3.4.1 Pure Breed With Lower Genetic Diversity (Breed_B)

**FIGURE 5:**
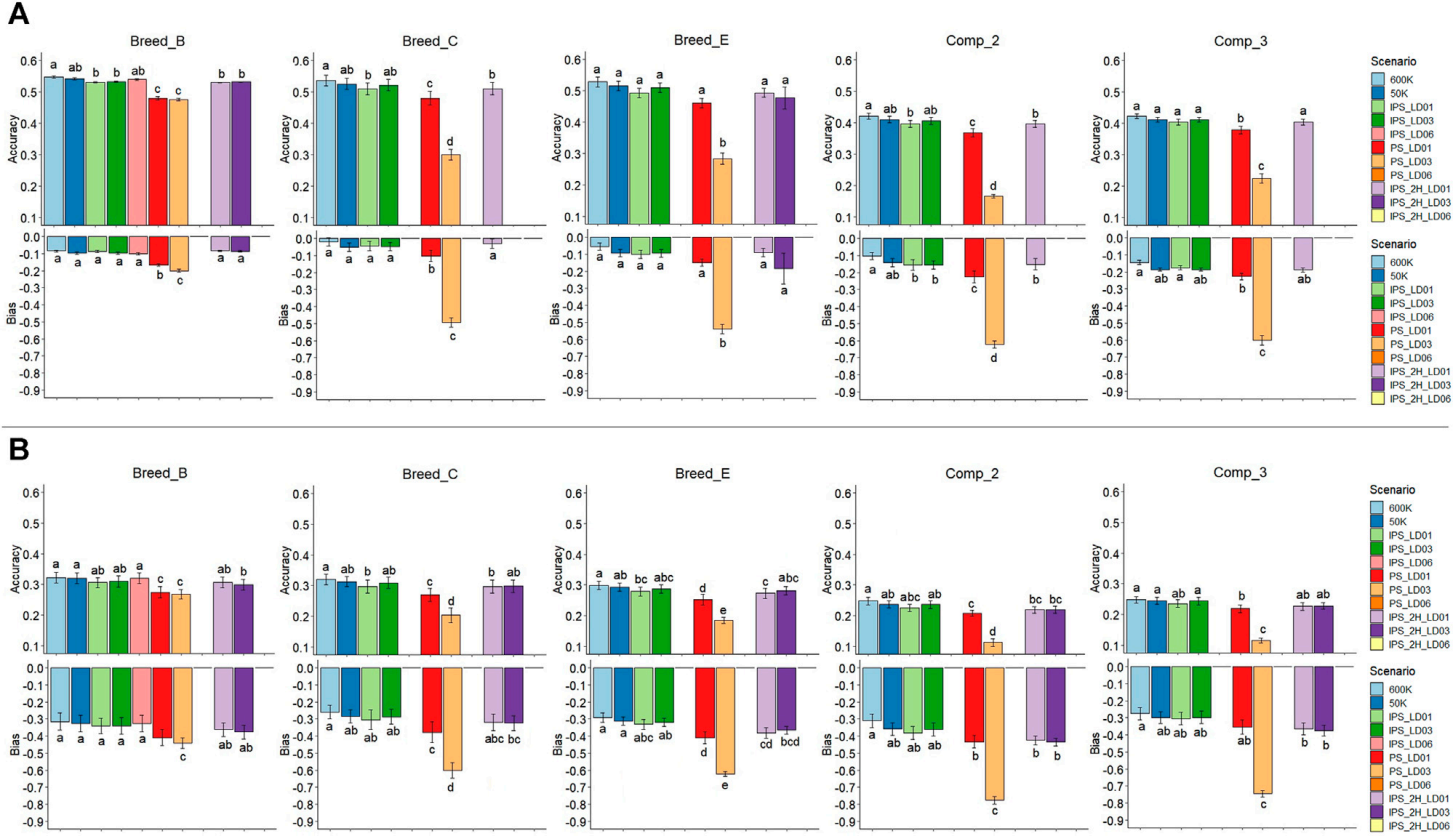
Accuracies and bias of genomic predictions based on individual SNPs and haplotypes for the simulations of traits with moderate **(A)** and low **(B)** heritability (0.30 and 0.10, respectively). Breed_B, Breed_C, and Breed_E: simulated pure breeds with different genetic backgrounds; Comp_2 and Comp_3: composite breeds from two and three pure breeds, respectively. 600 K: high-density panel; 50 K: medium-density panel; IPS_LD01, IPS_LD03, and IPS_LD06: independent and pseudo-SNPs from blocks with LD thresholds of 0.1, 0.3, and 0.6, respectively, in a single genomic relationship matrix; PS_LD01, PS_LD03, and PS_LD06: only pseudo-SNPs from blocks with LD threshold of 0.1, 0.3, and 0.6, respectively; and IPS_2H_LD01, IPS_2H_LD03, and IPS_2H_LD06: independent and pseudo-SNPs from blocks with LD thresholds of 0.1, 0.3, and 0.6, respectively, in two genomic relationship matrices. Zero values for both accuracies and bias mean no results were obtained, due to poor quality of genomic information or no convergence of the genomic prediction models. The same lower-case letters mean no statistical difference comparing genomic prediction methods within population at 5% significance level based on the Tukey test.

The average accuracy for GEBVs based on individual SNPs in the Breed_B was 0.54 and 0.55 for the 50 and 600 K panels, respectively, whereas it varied from 0.48 (pseudo-SNPs from blocks with an LD threshold of 0.3, PS_LD03) to 0.54 (independent SNPs and pseudo-SNPs from blocks with an LD threshold of 0.6, IPS_LD06) using haplotypes (Figure 5A, Supplementary Material S7). In general, genomic predictions that used pseudo-SNPs and independent SNPs in one or two relationship matrices did not statistically differ from those with SNPs in the 50 and 600 K panels. Using only pseudo-SNPs in the genomic predictions showed significantly lower accuracy than all other methods, when considering an LD threshold equal to 0.1 and 0.3 to create the blocks (PS_LD01 and PS_LD03, respectively). No predictions with PS_LD06 and IPS_2H_LD06 (independent SNPs and pseudo-SNPs from blocks with an LD threshold of 0.6 in two relationship matrices) were performed due to the low correlations observed between off-diagonal elements in $A_{22}$ and G constructed with only pseudo-SNPs from haploblocks with an LD threshold of 0.6 (Supplementary Material S8). The average GEBV bias was equal to −0.09 and −0.08 for the 50 and 600 K SNP panels, respectively, whereas it ranged between −0.20 (PS_LD03) and −0.08 (IPS_2H_LD01) with haplotypes. No statistical differences were observed in the average bias when the two SNP panel densities or the independent and pseudo-SNP in one or two relationship matrices were used. PS_LD01 and PS_LD03 generated statistically more biased GEBVs than all the other scenarios.

#### 3.4.2 Pure Breed With Medium-Size Founder Population and Moderate Genetic Diversity (Breed_C)

The average accuracy observed in the Breed_C was equal to 0.53 and 0.54 with the 50 and 600 K, respectively, while with haplotypes, it ranged from 0.25 (PS_LD03) to 0.52 (IPS_LD03) (Figure 5A, Supplementary Material S7). Similar to Breed_B, the PS_LD01 and PS_LD03 models yielded statistically less accurate GEBVs than all the other models, with PS_LD03 being the worst one. Fitting pseudo-SNPs and independent SNPs in one or two relationship matrices did not have statistical differences when compared with individual-SNP predictions. The IPS_2H_LD03 scenario did not converge during the genetic parameter estimation, and no pseudo-SNPs were generated for any haplotype method that used an LD threshold of 0.6 (IPS_LD06, PS_LD06, and IPS_2H_LD06). Consequently, no results were obtained for these scenarios. Average GEBV bias equal to −0.05 and −0.02 were observed for the 50 and 600 K SNP panels, whereas in the haplotype-based predictions, it ranged from −0.49 (PS_LD03) to −0.03 (IPS_2H_LD01). PS_LD01 and PS_LD03 were statistically more biased than all the other scenarios (statistically similar among them).

#### 3.4.3 Pure Breed With Larger Founder Population and Moderate Genetic Diversity (Breed_E)

The average accuracy was equal to 0.52 and 0.53 for the 50 and 600 K SNP panel, respectively, while the haplotype-based approach yielded accuracy varying between 0.28 (PS_LD03) and 0.51 (IPS_LD03) in Breed_E (Figure 5A, Supplementary Material S7). Using only pseudo-SNPs from haplotype blocks with an LD threshold of 0.3 (PSLD03) yielded the less accurate genomic predictions, being statistically lower than all the other models (with similar accuracy among them). No blocks with an LD threshold equal to 0.6 were created in this population, and therefore, no predictions were obtained with the models that would use pseudo-SNPs from these blocks. For the GEBV bias, averages of −0.09 and −0.06 were observed for the 50 and 600 K panels, respectively, ranging from −0.53 (PS_LD03) to −0.09 (IPS_2H_LD01) when haplotypes were fitted. Similar to the accuracy findings, the PSLD03 showed statistically lower average GEBV bias of prediction compared to all other models, showing the more biased predictions.

#### 3.4.4 Composite Breed From Two Populations With High Genetic Diversity (Comp_2)

The average accuracy for the 50 and 600 K SNP panels in Comp_2 were 0.41 and 0.42, respectively, with haplotype-based predictions ranging from 0.17 (PSLD03) to 0.41 (IPS_LD03) (Figure 5A, Supplementary Material S7). As observed in the pure breeds, there were no statistical differences between the predictions with SNPs based on both SNP density panels and the scenarios that fitted pseudo-SNPs and independent SNPs in one or two relationship matrices. Using only pseudo-SNPs to create the G matrix also provided statistically lower accuracy, with PS_LD03 yielding the worst results. No predictions were made with IPS_2H_LD03 in this population because of convergence problems during the genetic parameter estimation process. No pseudo-SNPs were obtained with the LD threshold of 0.6 and, consequently, no subsequent genomic prediction results. Average GEBV bias of −0.14 and −0.10 was observed for the 50 and 600 K SNP panels, respectively, while the average GEBV bias ranged from −0.62 (PS_LD03) to −0.15 (IPS_2H_LD01) when fitting haplotypes. Statistically, more biased predictions were obtained only when pseudo-SNPs from haplotype blocks with an LD threshold of 0.3 were used (PS_LD03).

#### 3.4.5 Composite Breed From Three Populations With High Genetic Diversity (Comp_3)

The average accuracy for the 50 and 600 K SNP panels were 0.41 and 0,42, respectively, and with haplotype-based predictions, they ranged from 0.22 (PS_LD03) to 0.41 (IPS_LD03) (Figure 5A, Supplementary Material S7). The PS_LD01 and PS_LD03 scenarios yielded statistically lower accuracy than all the other methods (statistically similar among them). Similarly to Comp_2, no genomic predictions were performed for the IPS_2H_LD03 and models fitting pseudo-SNPs from blocks with an LD threshold of 0.6. The average GEBV bias was −0.19 and −0.14 for the 50 and 600 K SNP panels, respectively, and ranged from −0.60 (PS_LD03) to −0.18 (IPS_LD01) for the haplotype-based predictions. Using only pseudo-SNPs from LD blocks constructed based on an LD threshold of 0.3 resulted in more biased GEBV predictions for the Comp_3 population.

### 3.5 Accuracy and Bias of Genomic Predictions: Low Heritability Trait

The effects of fitting haplotypes in the genomic predictions under the LH2 scenarios were similar to those observed in the MH2 scenarios for all populations, with also similar average results (Figure 5B and Supplementary Material S9). Therefore, the interpretations of the results for MH2 can be extended to the LH2 scenario, in which the worst results were observed for the PS_LD03 and similar accuracy and bias using SNPs or haplotypes (with independent SNPs) were observed. The GEBVs from the LH2 scenarios were less accurate and more biased than those from the MH2 scenarios within populations (e.g., lower accuracy and greater bias in LH2 within Breed_B), as would be expected due to the lower heritability of the trait. No GEBV predictions were made for the PS_LD06 and IPS_2H_LD06 for Breed_B due to the low correlation between the off-diagonal elements of the $A_{22}$ and G created with pseudo-SNPs from blocks with an LD threshold of 0.6 (Supplementary Material S10). No results for all scenarios fitting pseudo-SNPs from blocks with an LD threshold of 0.6 were obtained for Breed_C, Breed_E, Comp_2, and Comp_3 because no blocks were created based on this threshold.

## 4. Discussion

We hypothesized that the predicted GEBV in populations with higher genetic diversity, such as composite sheep breeds (e.g., Kijas et al., 2012; Brito et al., 2017b; Oliveira et al., 2020), could benefit from the use of haplotype-based rather than SNP-based genomic predictions, by obtaining GEBVs with higher accuracy and lower bias of prediction. Therefore, we investigated the impact of including haplotype information in ssGBLUP for populations with high genetic diversity, assessed based on the Ne metric, and different genetic background. Furthermore, we evaluated the performance of haplotype-based models by fitting the haplotypes as pseudo-SNPs in different ways under the ssGBLUP framework. For that, we considered only pseudo-SNPs to construct the genomic relationships and also two different relationship matrices (i.e., derived from individual SNPs and pseudo-SNPs from haplotype blocks), assuming no correlation between them. To evaluate our hypothesis, simulated data was used to calculate the true accuracy and bias of genomic predictions for simulated traits with moderate and low heritability level. These two sets of heritability levels comprise the major part of traits of interest in livestock breeding programs (e.g., growth, carcass, feed efficiency, reproductive performance, disease resistance, overall resilience).

### 4.1 Genetic Diversity and Genetic Parameters

The genetic diversity and variance components were assessed in the subsets of the data used for the predictions to verify the consistency of the initial simulation parameters. In addition to the first three recent Ne idealized at the beginning of this study (100, 250, and 500), several other genetic diversity measures were obtained after the simulation process was finalized, which are measures of recent Ne (until five generations ago) based on LD (Ne_LD_) and on realized inbreeding (Ne_Inb_) (Table 1 and Supplementary Materials S3, S4). Ne_LD_ would be more useful in the absence of accurate pedigree information, as it relies on the $E\left(r^{2}\right)$ estimation in a pre-defined chromosomic segment size and was proposed for simpler population structures (e.g., random mating and no selection; Sved, 1971). However, we also calculated Ne_Inb_ as an alternative indicator of Ne, because this estimate is based on the realized inbreeding and relies on the actual increase in population autozygosity (Falconer and Mackay, 1996).

One thousand and six hundred individuals from each one of the five populations (8,000 in total) were used to obtain the principal components (PCs) shown in Supplementary Material S2, which actually explained a small proportion of the overall variance (1.71 and 2.13% for the first two and first three PCs, respectively). McVean (2009) highlighted several situations that can affect the structure and spatial distribution of the PCA using SNPs (e.g., current and recurrent bottlenecks, admixture, waves of expansion, sample size) and potentially cause bias in the scatter with the first PCs, especially if they explain a little proportion of the overall variance. Rao (1964) also indicated that inferences about structural relationships using the first PCs are only recommended when they explain a substantial amount of variation, which was not our case. Also, Deniskova et al. (2016) found a sheep population with a lower Ne (176) more scattered in the first two PCs than populations with higher Ne (>500), indicating the need for a third PC to observe differences within the high genetically diverse, similar to what we observed in this current study. The authors mentioned that a small founder population could be the reason for the lower Ne in the more scattered population along the first two PCs, and the Breed_B in our study (lower Ne) also had the smallest founder population. Another important point to highlight is that when using commercially available SNP chips, there tends to be ascertainment bias in the design of the SNP panels, which then contributes to a greater differentiation among populations (depending if they contributed or not to the SNP panel design) and crossbred/composite animals tend to have greater SNP diversity and be more scattered in the plots. This does not tend to happen when using simulated datasets. In summary, as it is not recommended to make inferences with PCs that are not significant (Rao, 1964; McVean, 2009), the Ne should be used to make conclusions about the genetic diversity of the simulated populations, with the PCs used only for the illustration of the population structure.

Both Ne measures showed values close to those observed for some terminal and composite sheep breeds (125–974) as reported by Brito et al. (2017b), indicating that the simulation analyses resulted in datasets mimicking the genetic structure of commercial sheep populations. In addition to sheep, other species also present similar genetic diversity levels to some of the simulated populations used in this research, such as goats (Ne from 38 to 149; Brito et al., 2015), beef cattle (Ne from 153 to 220; Biegelmeyer et al., 2016), and dairy cattle (Ne from 58 to 120; Makanjuola et al., 2020). The genetic parameters estimated after the simulation process were similar and consistent among replicates across all recent populations used for the subsequent analyses in both scenarios (MH2 and LH2; Table 1 and Supplementary Materials S3, S4).

### 4.2 Statistics From Haplotype Blocks and Pseudo-SNPs

The differences observed on the haplotype block statistics across the simulated populations within LD thresholds and also across LD thresholds within populations are a consequence of the genetic events experienced by them. The number and size of the LD blocks can vary according to recombination hotspots and evolutionary events such as mutation, selection, migration, and random drift (McVean et al., 2004). In this context, a lower number of blocks with high LD thresholds would be expected in more genetically diverse populations, simply because in these populations, a large number of SNPs are expected to be excluded from all haploblocks, left to be considered as individual SNP effects. This was observed in Breed_B (less diverse, Ne ranging from 94 to 159) having a larger number of blocks not only when 0.6 was used as the LD threshold but also when the LD threshold was set to 0.3 in both MH2 and LH2 scenarios (Figures 3, 4 and Supplementary Materials S5, S6).

The average number of blocks was similar (LH2, Figure 4 and Supplementary Material S7) or even lower (MH2, Figure 3 and Supplementary Material S6) in Breed_B compared to the other populations when the LD threshold was set to 0.1. The Big-LD method used in this study defines the LD blocks by using weights estimated based on the number of SNPs from all possible overlapping intervals (Kim et al., 2018). Therefore, low LD thresholds could imply in similar intervals to derive the independent blocks regardless of the level of genetic diversity in populations derived from the same historical population (i.e., same species). When setting low LD thresholds to construct the LD-blocks, more intervals of linked SNPs are obtained as the number of blocks increase with less SNPs excluded (and vice versa). Therefore, this might explain the distribution of the number of blocks across populations with an LD threshold of 0.1. Consequently, a greater number of blocks are expected, as observed when comparing the number of blocks across LD thresholds (the number of blocks with an LD threshold of 0.1 > 0.3 > 0.6, Figures 3, 4 and Supplementary Materials S5, S6).

The number of blocked SNPs and pseudo-SNPs before and after QC in both MH2 and LH2 (Figures 3, 4 and Supplementary Materials S5, S6) is a function of the genetic diversity level of the populations. Longer blocks with many SNPs are expected in less genetically diverse populations (Hayes et al., 2003; Villumsen et al., 2009; Hess et al., 2017) likely due to selection and inbreeding, whereas more pseudo-SNPs (unique haplotypes) are expected in more genetically diverse populations (Teissier et al., 2020), when the single SNPs out of the LD-clusters are not considered as a block, following the standard definition of haplotype block (Gabriel et al., 2002). However, this also depends on the LD threshold used to create the haplotype blocks, as this pattern was clear only when LD was greater than 0.1.

Independently of the LD level used to create the blocks, the relative reduction in the number of pseudo-SNPs after QC was greater on the less genetically diverse population, with approximately 40% in Breed_B when the LD threshold was set to 0.6. The greatest reduction of pseudo-SNPs in populations with less genetic diversity was due to the low frequency of the haplotypes in this research, which agrees with the literature [e.g., based on simulated data (Villumsen et al., 2009); in dairy cattle populations (Hess et al., 2017; Karimi et al., 2018); and in dairy goats (Teissier et al., 2020)].

The additional computing time needed for genotype phasing, creating the haplotype blocks and the covariates for the models (Feitosa et al., 2019; Teissier et al., 2020), and running the genomic predictions (Cuyabano et al., 2015; Hess et al., 2017) have been indicated as the main drawbacks for the use of haplotypes in routine genomic predictions. In this study, the maximum additional computing time observed was approximately 7 h (23,663.6 s, Breed_B with LD equal to 0.1 under the LH2 scenario—Figure 4A and Supplementary Material S6). Hess et al. (2017) used marker effect models under Bayesian approaches and observed additional time of up to 27.2 h for predictions with haplotypes derived from 37 K SNPs with training and validation populations of about 30,000 dairy cattle individuals. Cuyabano et al. (2015) reported that genomic predictions using Bayesian approaches and haplotypes took approximately from 1 to 46 h, depending on the number of previously associated SNPs included in the GEBV predictions (1–50 K, respectively), with approximately 4,000 individuals in the training and validation populations. Differently from these studies, we used the ssGBLUP method, which showed consistent time for the predictions in the 50 K SNP panel or when fitting haplotypes (as pseudo-SNPs) in the same G matrix. This was likely observed because the GEBVs are estimated directly based on the G matrix and the number of pseudo-SNPs added to the non-blocked SNPs (Figures 3, 4 and Supplementary Materials S5, S6) was not large enough to require longer time to create the genomic relationship matrices. As we calculated GEBVs for more than 62,000 individuals (genotyped and non-genotyped) using haplotype information with a relatively low increase of time, ssGBLUP is a feasible alternative for that purpose.

Interestingly, our results suggest that the computing time to obtain pseudo-SNPs in less genetically diverse populations is higher than in more diverse populations. This could be because more diverse populations have a smaller number of intervals with a determined LD level than populations with low genetic diversity, implying in less iterations for the algorithm to create the haplotype blocks. The smaller number of candidate intervals to create the blocks, leading to a lower computing time, might also explain the differences observed when comparing the LD levels within populations, with the computing time being significantly greater with an LD threshold of 0.1, followed by 0.3 and 0.6 LD thresholds.

### 4.3 Accuracy and Bias of Genomic Predictions

Genomic predictions based on whole genome sequence (WGS) data could be more advantageous because all the causal mutations are expected to be included in the data. However, practical results have shown no increase in GEBV accuracy when using WGS over HD (Binsbergen et al., 2015; Ni et al., 2017) or even medium density (∼50 K) SNP panels (Frischknecht et al., 2018). HD SNP panels were developed to better capture the LD between SNPs and QTLs and thus improve the ability to detect QTLs and obtain more accurate GEBVs (Kijas et al., 2014), especially in more genetically diverse populations or even across-breed genomic predictions. However, the 50 K SNP panel has shown a similar predictive ability to the HD even in highly diverse populations as in sheep (Moghaddar et al., 2017). These findings corroborate with our results using the 50 K SNP panel, regardless of the trait heritability. This suggests that both SNP panels (i.e., 50 and 600 K) are sufficient to capture the genetic relationships of the individuals, which is the base of the genomic predictions based on the ssGBLUP method (Legarra et al., 2009; Aguilar et al., 2010; Lourenco et al., 2020). Therefore, we used the 50 K SNP panel for haplotype-based genomic predictions.

Genomic predictions are expected to be more accurate with haplotypes instead of individual SNPs mainly because they are expected to be in greater LD with the QTL than are individual markers (Calus et al., 2008; Villumsen et al., 2009; Cuyabano et al., 2014, 2015; Hess et al., 2017). In this context, Calus et al. (2008) and Villumsen et al. (2009) reported better results for the haplotype-based predictions of GEBVs than individual SNPs in simulated data, highlighting the possibility of improving both the accuracy and bias of genomic predictions. The Ne of the populations used by Calus et al. (2008) and Villumsen et al. (2009) is similar to the one in Breed_B (∼100). However, in this current study, haplotype-based models provided similar or lower accuracy and they were also similar or more biased than individual SNP-based models under both MH2 or LH2 scenarios (Figure 5 and Supplementary Materials S7, S9). This might be related to the LD level between SNP-QTL and haplotype-QTL and also the amount of information used to estimate the SNP and haplotype effects. Calus et al. (2008) and Villumsen et al. (2009) had fewer individuals (∼1,000), and their simulations were done with more general parameters compared to our study. The training set in this research for all populations was composed by 60,000 individuals with phenotypes, in which 8,000 of them were also genotyped. This amount of data is likely enough to estimate SNP effects and also the SNP-QTL LD properly. Thus, predictions with SNPs and haplotypes did not differ in some cases due to both of them capturing well the genetic relationships to achieve similar prediction results.

The correlations between off-diagonal, diagonal, and all elements in $A_{22}$ and G created with pseudo-SNPs and independent SNPs together were similar to fit only individual SNPs in both SNP panel densities for all LD thresholds and in all populations, regardless of the heritability (Supplementary Materials S8, S10). Furthermore, the average, maximum, and minimum values of the diagonal elements in G created when combining pseudo-SNPs and independent SNPs were also similar to using only individual SNPs for both SNP panel densities in all scenarios investigated. Therefore, combining haplotypes and SNPs in a single G matrix captured the same information as fitting only individual SNPs, and, consequently, resulting in similar GEBV predictions.

Another reason for the similar genomic predictions when fitting individual SNPs and haplotypes might be the absence of or negligible epistatic interaction effects between SNP loci within haplotype blocks. In humans, a species with high Ne (Park, 2011), Liang et al. (2020) showed that epistasis was the reason for increased accuracy with haplotypes over individual SNPs for health traits. In other words, a similar accuracy between SNPs and haplotypes was observed when there was negligible epistasis effect. The same authors also pointed out that predictions using haplotypes might only be worse than fitting individual SNPs because of a possible “haplotype loss,” which can happen when SNP effects are not accurately estimated by the haplotypes. As no epistatic effects are currently simulated by QMSim (Sargolzaei and Schenkel, 2009) and, therefore, were not simulated in the current study, different from our assumption that haplotypes could improve the predictions in more genetically diverse populations (Breed_C, Breed_E, Comp_2, and Comp_3), the accuracy and bias estimated based on haplotypes were similar or worse compared to fitting individual SNPs.

Many studies based on real datasets have shown small improvements in the performance of haplotype-based genomic predictions. For instance, Cuyabano et al. (2014) showed up to a 3.1% increase in the accuracy for milk protein when using LD-based haplotypes. Cuyabano et al. (2015) also obtained gains in accuracy of up to 1.3% using pre-selected SNPs associated with the trait combined with the haplotypes as covariates in the models for production, fertility, and health traits. Mucha et al. (2019) showed no differences in predictions with high-frequency haplotypes compared to SNPs when evaluating reproductive performance traits and somatic cell score in Polish dairy cattle. Additionally, Feitosa et al. (2019) obtained nearly the same accuracy and bias for meat fatty acid (MFA) traits in Nellore cattle when fitting individual SNPs or haplotypes. These findings indicate that, even in instances where haplotypes are better than SNPs, the improvements are negligible or small. However, considerable improvements in haplotype-based predictions have also been reported in the literature for relatively less polygenic traits with known major genes or when using biological information to construct the haplotype blocks. Won et al. (2020) reported a significant increase of 4.6% in GEBV accuracy with LD-clustering-based haplotypes for eye muscle area in Korean cattle. In Simmental cattle, Xu et al. (2020) reported increases of 9.8% in carcass weight when incorporating haplotype information based on SNPs from functionally related genomic regions. Teissier et al. (2020) reported an increase in accuracy of up to 22% when using haplotypes from fixed length or LD blocking strategies under an ssGBLUP setting. Based on these literature reports in livestock, it seems that haplotype predictions could provide better results when traits are oligogenic or affected by major genes, which are less common in livestock breeding goals. In addition, the presence of epistatic interactions in a real situation can also provide better results (Liang et al., 2020). In this sense, using biological information to create the blocks of linked markers to make haplotype predictions can be an alternative to improve the genomic predictions in genetically diverse livestock populations. Unfortunately, there are limited real datasets of enough size with both phenotypes and genotypes for populations with large Ne that could be used for validating our findings.

It is worth mentioning that haplotype-based models without including the independent SNPs (markers not assigned to any block) to create the genomic relationships always provided the worst results, regardless of the LD threshold to create the haploblocks (0.1, 0.3, and 0.6). These models were also less accurate and more biased in all the populations, regardless of the genetic diversity level and heritability (Figure 5 and Supplementary Materials S7, S9). The worst results were obtained when fitting only pseudo-SNPs from blocks with an LD threshold of 0.3 (PSLD03) and in more genetically diverse populations (Breed_C, Breed_E, Comp_2, and Comp_3). This might have occurred because fitting only pseudo-SNPs from the haploblocks with two or more SNPs is not enough to consider all the important chromosomic regions influencing the trait of interest. The number of blocks, blocked SNPs, and pseudo-SNPs that were used to make the predictions were significantly lower with the LD level of 0.3 compared to 0.1 in both simulations (Figures 3, 4 and Supplementary Materials S5, S6), with this being likely the reason for the lowest accuracy and largest bias observed for PS_LD03. In this context, increasing the LD threshold to create the haploblocks have hampered the prediction with only haplotypes because a larger number of genomic markers were not considered to make the predictions. However, increasing the LD threshold to create the blocks and using the non-clustered SNPs together with the pseudo-SNPs did not affect the prediction results, presenting similar GEBV accuracies and bias compared to SNP-based predictions. In addition, the main differences in the properties of the G matrix were observed when only pseudo-SNPs from haploblocks with bigger LD thresholds were used, with lower correlations between off-diagonal and all elements in the $A_{22}$ and G matrices and differences in the maximum and minimum values of the diagonal elements of the G (Supplementary Materials S8, S10). Therefore, independently of the LD threshold used to create the haploblocks, we recommend using the non-clustered SNPs with pseudo-SNPs from multi-marker haploblocks to make haplotype-based predictions, as well as in genome-wide association studies (GWAS) using haplotypes, because these variants may play an important role.

Separating the independent and pseudo-SNPs in two different random effects, with no shared covariances structures, did not significantly impact the genomic predictions, but had a computational cost. The genetic parameter estimation and GEBV prediction required more computing time using these two genetic components in the model, with more iterations and greater time in each iteration than the other models (data not shown), sometimes leading to no convergence of the solutions (IPS_2H_LD03 in the Breed_C, Comp_2, and Comp_3 under MH2). The model with pseudo-SNPs and independent SNPs in two genetic components is more complex, and the convergence difficulty might suggest poor model parametrization, potentially because the random effects were assumed to be uncorrelated. This fact can be confirmed by high correlations (above than 0.90) between the inverted H matrices with non-clustered SNPs and pseudo-SNPs (data not shown). Although increased computational time was a common problem in both heritability levels, convergence was achieved in all analyses with low heritability. Our findings suggest that a single G matrix with individual SNPs is enough to capture the QTL variation, regardless of the genetic diversity and heritability. Nonetheless, using two uncorrelated genetic components can be useful in other situations such as fitting SNPs and structural variants (e.g., copy number variation—CNVs) in the same model.

## 5 Conclusion

Haplotype-based models did not improve the performance of genomic prediction of breeding values in genetically diverse populations (assumed as Ne > 150) under ssGBLUP settings. A medium-density 50 K SNP panel provided similar results to the high-density panel for the genomic predictions using individual SNPs or haplotypes, regardless of the heritability and genetic diversity levels. ssGBLUP can be used to predict breeding values for both genotyped and non-genotyped individuals using haplotype information in large datasets with no increase in computing time when fitting a single genomic relationship matrix.

## Data Availability

The simulated datasets used and the pipelines developed to carry out this research are available upon request.
