# Supplementary material for: A Comprehensive Comparison of Haplotype-Based Single-Step Genomic Predictions in Livestock Populations With Different Genetic Diversity Levels: A Simulation Study
Source: Front Genet. 2021 Oct 14;12:729867. doi: 10.3389/fgene.2021.729867 (PMC8551834; doi:10.3389/fgene.2021.729867)

# Statistics from blocks and pseudo-SNPs: low heritability

## [Statistics from blocks and pseudo-SNPs: low heritability](#content)

- [Summary](#summary)

Andre Araujo

22/05/2021

# Summary

In this file is shown the values for each repetition and also the average and standard error mean for statistics related to the haplotype block strategies for each simulated population used on the research entitled A comprehensive comparison of single-step genomic BLUP approaches fitting SNPs or haplotypes in populations with different genetic diversity levels: a simulation study, by Araujo et al.

- The statistics are:
  - 1. Number of blocks
    2. Number of blocked SNPs;
    3. Number of pseudo-SNPs;
    4. Number of pseudo-SNPs after quality control (QC);
    5. Number of non-blocked plus pseudo-SNPs after QC.
    6. Duration time to get the pseudo SNPs

| Table 1. Number of blocks created with LD threshold of 0.1. | | | | | | | |
| --- | --- | --- | --- | --- | --- | --- | --- |
| **Population** | **rep_1** | **rep_2** | **rep_3** | **rep_4** | **rep_5** | **average** | **sem^1^** |
| Breed_B | 7643 | 7418 | 7768 | 7589 | 7717 | 7627.0 | 60.56484 |
| Breed_C | 7582 | 6966 | 7091 | 7194 | 7222 | 7211.0 | 103.07182 |
| Breed_E | 7799 | 7116 | 7072 | 6941 | 7228 | 7231.2 | 149.20436 |
| Comp_2 | 8190 | 7996 | 8399 | 8258 | 7857 | 8140.0 | 96.00260 |
| Comp_3 | 8614 | 6983 | 8470 | 8795 | 8603 | 8293.0 | 331.55497 |
| ^1^ Standard error mean. |  |  |  |  |  |  |  |
| ^2^ rep_1 to rep_5: repetitions. |  |  |  |  |  |  |  |

| Table 2. Number of blocks created with LD threshold of 0.3. | | | | | | | |
| --- | --- | --- | --- | --- | --- | --- | --- |
| **Population** | **rep_1** | **rep_2** | **rep_3** | **rep_4** | **rep_5** | **average** | **sem^1^** |
| Breed_B | 3195 | 2469 | 3685 | 2346 | 3555 | 3050.0 | 274.99564 |
| Breed_C | 719 | 975 | 769 | 723 | 939 | 825.0 | 54.89627 |
| Breed_E | 576 | 1254 | 1281 | 1301 | 938 | 1070.0 | 140.21377 |
| Comp_2 | 248 | 216 | 331 | 329 | 199 | 264.6 | 27.83631 |
| Comp_3 | 385 | 861 | 342 | 588 | 514 | 538.0 | 91.98641 |
| ^1^ Standard error mean. |  |  |  |  |  |  |  |
| ^2^ rep_1 to rep_5: repetitions. |  |  |  |  |  |  |  |

| Table 3. Number of blocks created with LD threshold of 0.6. | | | | | | | |
| --- | --- | --- | --- | --- | --- | --- | --- |
| **Population** | **rep_1** | **rep_2** | **rep_3** | **rep_4** | **rep_5** | **average** | **sem^1^** |
| Breed_B | 10 | 5 | 17 | 3 | 18 | 10.6 | 3.043025 |
| Breed_C | 0 | 0 | 0 | 0 | 0 | 0.0 | 0.000000 |
| Breed_E | 0 | 0 | 0 | 0 | 0 | 0.0 | 0.000000 |
| Comp_2 | 0 | 0 | 0 | 0 | 0 | 0.0 | 0.000000 |
| Comp_3 | 0 | 0 | 0 | 0 | 0 | 0.0 | 0.000000 |
| ^1^ Standard error mean. |  |  |  |  |  |  |  |
| ^2^ rep_1 to rep_5: repetitions. |  |  |  |  |  |  |  |

| Table 4. Number of SNPs within blocks (blocked) with LD threshold of 0.1. | | | | | | | |
| --- | --- | --- | --- | --- | --- | --- | --- |
| **Population** | **rep_1** | **rep_2** | **rep_3** | **rep_4** | **rep_5** | **average** | **sem^1^** |
| Breed_B | 17823 | 17177 | 18219 | 17401 | 18119 | 17747.8 | 201.4394 |
| Breed_C | 17109 | 15911 | 16027 | 16197 | 16467 | 16342.2 | 213.2985 |
| Breed_E | 17542 | 16296 | 16301 | 15855 | 16467 | 16492.2 | 281.4064 |
| Comp_2 | 18249 | 17804 | 18713 | 18430 | 17495 | 18138.2 | 218.3134 |
| Comp_3 | 19146 | 15939 | 18834 | 19605 | 19232 | 18551.2 | 664.5049 |
| ^1^ Standard error mean. |  |  |  |  |  |  |  |
| ^2^ rep_1 to rep_5: repetitions. |  |  |  |  |  |  |  |

| Table 5. Number of SNPs within blocks (blocked) with LD threshold of 0.3. | | | | | | | |
| --- | --- | --- | --- | --- | --- | --- | --- |
| **Population** | **rep_1** | **rep_2** | **rep_3** | **rep_4** | **rep_5** | **average** | **sem^1^** |
| Breed_B | 7321 | 5627 | 8435 | 5338 | 8161 | 6976.4 | 638.5443 |
| Breed_C | 1680 | 2265 | 1803 | 1695 | 2197 | 1928.0 | 125.9659 |
| Breed_E | 1347 | 2887 | 2960 | 3022 | 2173 | 2477.8 | 321.5375 |
| Comp_2 | 568 | 481 | 781 | 750 | 455 | 607.0 | 67.5374 |
| Comp_3 | 889 | 1983 | 786 | 1338 | 1186 | 1236.4 | 211.4026 |
| ^1^ Standard error mean. |  |  |  |  |  |  |  |
| ^2^ rep_1 to rep_5: repetitions. |  |  |  |  |  |  |  |

| Table 6. Number of SNPs within blocks (blocked) with LD threshold of 0.6. | | | | | | | |
| --- | --- | --- | --- | --- | --- | --- | --- |
| **Population** | **rep_1** | **rep_2** | **rep_3** | **rep_4** | **rep_5** | **average** | **sem^1^** |
| Breed_B | 25 | 11 | 41 | 7 | 42 | 25.2 | 7.296575 |
| Breed_C | 0 | 0 | 0 | 0 | 0 | 0.0 | 0.000000 |
| Breed_E | 0 | 0 | 0 | 0 | 0 | 0.0 | 0.000000 |
| Comp_2 | 0 | 0 | 0 | 0 | 0 | 0.0 | 0.000000 |
| Comp_3 | 0 | 0 | 0 | 0 | 0 | 0.0 | 0.000000 |
| ^1^ Standard error mean. |  |  |  |  |  |  |  |
| ^2^ rep_1 to rep_5: repetitions. |  |  |  |  |  |  |  |

| Table 7. Number of pseudo-SNPs from blocks with LD threshold of 0.1. | | | | | | | |
| --- | --- | --- | --- | --- | --- | --- | --- |
| **Population** | **rep_1** | **rep_2** | **rep_3** | **rep_4** | **rep_5** | **average** | **sem^1^** |
| Breed_B | 46330 | 44359 | 47904 | 43381 | 48306 | 46056.0 | 964.0569 |
| Breed_C | 41580 | 39276 | 38808 | 39080 | 52471 | 42243.0 | 2604.4043 |
| Breed_E | 42531 | 41223 | 41338 | 39304 | 40828 | 41044.8 | 519.8028 |
| Comp_2 | 43288 | 42266 | 44865 | 44028 | 41364 | 43162.2 | 620.5506 |
| Comp_3 | 45362 | 40075 | 44545 | 47176 | 45964 | 44624.4 | 1215.5778 |
| ^1^ Standard error mean. |  |  |  |  |  |  |  |
| ^2^ rep_1 to rep_5: repetitions. |  |  |  |  |  |  |  |

| Table 8. Number of pseudo-SNPs from blocks with LD threshold of 0.3. | | | | | | | |
| --- | --- | --- | --- | --- | --- | --- | --- |
| **Population** | **rep_1** | **rep_2** | **rep_3** | **rep_4** | **rep_5** | **average** | **sem^1^** |
| Breed_B | 17572 | 13356 | 20304 | 12720 | 19774 | 16745.2 | 1584.4746 |
| Breed_C | 4072 | 5436 | 4364 | 4128 | 5392 | 4678.4 | 304.3593 |
| Breed_E | 3280 | 6944 | 7168 | 7308 | 5240 | 5988.0 | 772.7064 |
| Comp_2 | 1368 | 1084 | 1896 | 1728 | 1080 | 1431.2 | 166.1344 |
| Comp_3 | 2132 | 4808 | 1884 | 3124 | 2844 | 2958.4 | 514.7234 |
| ^1^ Standard error mean. |  |  |  |  |  |  |  |
| ^2^ rep_1 to rep_5: repetitions. |  |  |  |  |  |  |  |

| Table 9. Number of pseudo-SNPs from blocks with LD threshold of 0.6. | | | | | | | |
| --- | --- | --- | --- | --- | --- | --- | --- |
| **Population** | **rep_1** | **rep_2** | **rep_3** | **rep_4** | **rep_5** | **average** | **sem^1^** |
| Breed_B | 64 | 24 | 100 | 16 | 96 | 60 | 17.52712 |
| Breed_C | 0 | 0 | 0 | 0 | 0 | 0 | 0.00000 |
| Breed_E | 0 | 0 | 0 | 0 | 0 | 0 | 0.00000 |
| Comp_2 | 0 | 0 | 0 | 0 | 0 | 0 | 0.00000 |
| Comp_3 | 0 | 0 | 0 | 0 | 0 | 0 | 0.00000 |
| ^1^ Standard error mean. |  |  |  |  |  |  |  |
| ^2^ rep_1 to rep_5: repetitions. |  |  |  |  |  |  |  |

| Table 10. Number of pseudo-SNPs after quality control from blocks with LD threshold of 0.1. | | | | | | | |
| --- | --- | --- | --- | --- | --- | --- | --- |
| **Population** | **rep_1** | **rep_2** | **rep_3** | **rep_4** | **rep_5** | **average** | **sem^1^** |
| Breed_B | 35933 | 34837 | 36461 | 35578 | 36290 | 35819.8 | 288.9120 |
| Breed_C | 35399 | 32935 | 33248 | 33659 | 33896 | 33827.4 | 426.3258 |
| Breed_E | 36384 | 33547 | 33668 | 32725 | 34071 | 34079.0 | 616.3745 |
| Comp_2 | 37793 | 36981 | 38701 | 38001 | 36289 | 37553.0 | 418.3950 |
| Comp_3 | 39529 | 32728 | 38838 | 40267 | 39732 | 38218.8 | 1391.6504 |
| ^1^ Standard error mean. |  |  |  |  |  |  |  |
| ^2^ rep_1 to rep_5: repetitions. |  |  |  |  |  |  |  |

| Table 11. Number of pseudo-SNPs after quality control from blocks with LD threshold of 0.3. | | | | | | | |
| --- | --- | --- | --- | --- | --- | --- | --- |
| **Population** | **rep_1** | **rep_2** | **rep_3** | **rep_4** | **rep_5** | **average** | **sem^1^** |
| Breed_B | 14732 | 11421 | 16930 | 10862 | 16303 | 14049.6 | 1243.1918 |
| Breed_C | 3582 | 4795 | 3816 | 3584 | 4604 | 4076.2 | 259.7542 |
| Breed_E | 2887 | 6041 | 6218 | 6320 | 4559 | 5205.0 | 661.7677 |
| Comp_2 | 1184 | 1021 | 1669 | 1594 | 951 | 1283.8 | 147.3741 |
| Comp_3 | 1887 | 4107 | 1631 | 2803 | 2469 | 2579.4 | 434.4322 |
| ^1^ Standard error mean. |  |  |  |  |  |  |  |
| ^2^ rep_1 to rep_5: repetitions. |  |  |  |  |  |  |  |

| Table 12. Number of pseudo-SNPs after quality control from blocks with LD threshold of 0.6. | | | | | | | |
| --- | --- | --- | --- | --- | --- | --- | --- |
| **Population** | **rep_1** | **rep_2** | **rep_3** | **rep_4** | **rep_5** | **average** | **sem^1^** |
| Breed_B | 37 | 16 | 66 | 12 | 69 | 40 | 12.01249 |
| Breed_C | 0 | 0 | 0 | 0 | 0 | 0 | 0.00000 |
| Breed_E | 0 | 0 | 0 | 0 | 0 | 0 | 0.00000 |
| Comp_2 | 0 | 0 | 0 | 0 | 0 | 0 | 0.00000 |
| Comp_3 | 0 | 0 | 0 | 0 | 0 | 0 | 0.00000 |
| ^1^ Standard error mean. |  |  |  |  |  |  |  |
| ^2^ rep_1 to rep_5: repetitions. |  |  |  |  |  |  |  |

| Table 13. Number of non-blocked SNPs plus pseudo-SNPs after quality control from blocks with LD threshold of 0.1. | | | | | | | |
| --- | --- | --- | --- | --- | --- | --- | --- |
| **Population** | **rep_1** | **rep_2** | **rep_3** | **rep_4** | **rep_5** | **average** | **sem^1^** |
| Breed_B | 64937 | 64487 | 65069 | 65004 | 64998 | 64899.0 | 105.0985 |
| Breed_C | 65117 | 63851 | 64048 | 64289 | 64256 | 64312.2 | 216.0910 |
| Breed_E | 65669 | 64078 | 64194 | 63697 | 64431 | 64413.8 | 335.4623 |
| Comp_2 | 66371 | 66004 | 66815 | 66398 | 65621 | 66241.8 | 201.4436 |
| Comp_3 | 67210 | 63616 | 66831 | 67489 | 67327 | 66494.6 | 727.7713 |
| ^1^ Standard error mean. |  |  |  |  |  |  |  |
| ^2^ rep_1 to rep_5: repetitions. |  |  |  |  |  |  |  |

| Table 14. Number of non-blocked SNPs plus pseudo-SNPs after quality control from blocks with LD threshold of 0.3. | | | | | | | |
| --- | --- | --- | --- | --- | --- | --- | --- |
| **Population** | **rep_1** | **rep_2** | **rep_3** | **rep_4** | **rep_5** | **average** | **sem^1^** |
| Breed_B | 54238 | 52621 | 55322 | 52351 | 54969 | 53900.2 | 604.74502 |
| Breed_C | 48729 | 49357 | 48840 | 48716 | 49234 | 48975.2 | 133.94529 |
| Breed_E | 48367 | 49981 | 50085 | 50125 | 49213 | 49554.2 | 340.26319 |
| Comp_2 | 47443 | 47367 | 47715 | 47671 | 47323 | 47503.8 | 79.89393 |
| Comp_3 | 47825 | 48951 | 47672 | 48292 | 48110 | 48170.0 | 223.10020 |
| ^1^ Standard error mean. |  |  |  |  |  |  |  |
| ^2^ rep_1 to rep_5: repetitions. |  |  |  |  |  |  |  |

| Table 15. Number of non-blocked SNPs plus pseudo-SNPs after quality control from blocks with LD threshold of 0.6. | | | | | | | |
| --- | --- | --- | --- | --- | --- | --- | --- |
| **Population** | **rep_1** | **rep_2** | **rep_3** | **rep_4** | **rep_5** | **average** | **sem^1^** |
| Breed_B | 46839 | 46832 | 46852 | 46832 | 46854 | 46841.8 | 4.758151 |
| Breed_C | 46827 | 46827 | 46827 | 46827 | 46827 | 46827.0 | 0.000000 |
| Breed_E | 46827 | 46827 | 46827 | 46827 | 46827 | 46827.0 | 0.000000 |
| Comp_2 | 46827 | 46827 | 46827 | 46827 | 46827 | 46827.0 | 0.000000 |
| Comp_3 | 46827 | 46827 | 46827 | 46827 | 46827 | 46827.0 | 0.000000 |
| ^1^ Standard error mean. |  |  |  |  |  |  |  |
| ^2^ rep_1 to rep_5: repetitions. |  |  |  |  |  |  |  |

| Table 16. Duration time to create pseudo-SNPs from blocks with LD threshold of 0.1. | | | | | | | |
| --- | --- | --- | --- | --- | --- | --- | --- |
| **Population** | **rep_1** | **rep_2** | **rep_3** | **rep_4** | **rep_5** | **average** | **sem^1^** |
| Breed_B | 16641 | 18656 | 18803 | 23874 | 21771 | 19949.0 | 1277.759 |
| Breed_C | 17914 | 19338 | 25758 | 20232 | 35076 | 23663.6 | 3147.809 |
| Breed_E | 18989 | 21283 | 15297 | 12868 | 18060 | 17299.4 | 1465.422 |
| Comp_2 | 9844 | 11655 | 17207 | 25433 | 8991 | 14626.0 | 3056.963 |
| Comp_3 | 11357 | 15009 | 10938 | 20456 | 18424 | 15236.8 | 1883.915 |
| ^1^ Standard error mean. |  |  |  |  |  |  |  |
| ^2^ rep_1 to rep_5: repetitions. |  |  |  |  |  |  |  |

| Table 17. Duration time to create pseudo-SNPs from blocks with LD threshold of 0.3. | | | | | | | |
| --- | --- | --- | --- | --- | --- | --- | --- |
| **Population** | **rep_1** | **rep_2** | **rep_3** | **rep_4** | **rep_5** | **average** | **sem^1^** |
| Breed_B | 2422 | 1952 | 3217 | 1824 | 2920 | 2467.0 | 269.11039 |
| Breed_C | 1096 | 1138 | 966 | 1085 | 889 | 1034.8 | 46.31782 |
| Breed_E | 800 | 1191 | 1133 | 1833 | 917 | 1174.8 | 179.18884 |
| Comp_2 | 722 | 713 | 728 | 1390 | 710 | 852.6 | 134.38809 |
| Comp_3 | 784 | 1023 | 771 | 1270 | 727 | 915.0 | 102.69615 |
| ^1^ Standard error mean. |  |  |  |  |  |  |  |
| ^2^ rep_1 to rep_5: repetitions. |  |  |  |  |  |  |  |

| Table 18. Duration time to create pseudo-SNPs from blocks with LD threshold of 0.6. | | | | | | | |
| --- | --- | --- | --- | --- | --- | --- | --- |
| **Population** | **rep_1** | **rep_2** | **rep_3** | **rep_4** | **rep_5** | **average** | **sem^1^** |
| Breed_B | 616 | 670 | 637 | 693 | 645 | 652.2 | 13.36937 |
| Breed_C | 587 | 645 | 670 | 715 | 614 | 646.2 | 22.18423 |
| Breed_E | 618 | 664 | 595 | 648 | 606 | 626.2 | 12.94759 |
| Comp_2 | 630 | 655 | 687 | 749 | 595 | 663.2 | 26.21908 |
| Comp_3 | 659 | 720 | 632 | 844 | 607 | 692.4 | 42.30910 |
| ^1^ Standard error mean. |  |  |  |  |  |  |  |
| ^2^ rep_1 to rep_5: repetitions. |  |  |  |  |  |  |  |


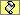

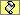

Supplement: Supplementary file 2 [file Table6.DOC]
