# Supplementary material for: A Comprehensive Comparison of Haplotype-Based Single-Step Genomic Predictions in Livestock Populations With Different Genetic Diversity Levels: A Simulation Study
Source: Front Genet. 2021 Oct 14;12:729867. doi: 10.3389/fgene.2021.729867 (PMC8551834; doi:10.3389/fgene.2021.729867)

# Ne and genetic parameters for the simulation with moderate heritability

## [Ne and genetic parameters for the simulation with moderate heritability](#content)

- [Summary](#summary)

Andre Araujo

20/05/2021

# Summary

In this file is shown the values for each repetition and also the average and standard error mean for effective population size and variance components in the simulated populations used on the research entitled A comprehensive comparison of single-step genomic BLUP approaches fitting SNPs or haplotypes in populations with different genetic diversity levels: a simulation study, by Araujo et al.

| Table 1. Effective population size calculated based in the linkage disequilibrium (Ne_LD_). | | | | | | | |
| --- | --- | --- | --- | --- | --- | --- | --- |
| **Population** | **rep_1** | **rep_2** | **rep_3** | **rep_4** | **rep_5** | **average** | **sem^1^** |
| Breed_B | 100 | 93 | 117 | 116 | 123 | 110 | 6 |
| Breed_C | 378 | 362 | 362 | 394 | 401 | 379 | 8 |
| Breed_E | 344 | 352 | 373 | 360 | 367 | 359 | 5 |
| Comp_2 | 607 | 689 | 670 | 623 | 630 | 644 | 15 |
| Comp_3 | 462 | 328 | 576 | 478 | 485 | 466 | 40 |
| ^1^ Standard error mean. |  |  |  |  |  |  |  |
| ^2^ rep_1 to rep_5: repetitions. |  |  |  |  |  |  |  |

| Table 2. Effective population size calculated based in the realized inbreeding (Ne_Inb_). | | | | | | | |
| --- | --- | --- | --- | --- | --- | --- | --- |
| **Population** | **rep_1** | **rep_2** | **rep_3** | **rep_4** | **rep_5** | **average** | **sem^1^** |
| Breed_B | 170 | 151 | 262 | 180 | 184 | 190 | 17 |
| Breed_C | 269 | 277 | 192 | 279 | 283 | 260 | 15 |
| Breed_E | 176 | 192 | 217 | 186 | 190 | 192 | 6 |
| Comp_2 | 446 | 416 | 454 | 456 | 460 | 446 | 7 |
| Comp_3 | 446 | 249 | 624 | 456 | 460 | 447 | 53 |
| ^1^ Standard error mean. |  |  |  |  |  |  |  |
| ^2^ rep_1 to rep_5: repetitions. |  |  |  |  |  |  |  |

| Table 3. Additive genetic variances for the pedigree-based model. | | | | | | | |
| --- | --- | --- | --- | --- | --- | --- | --- |
| **Population** | **rep_1** | **rep_2** | **rep_3** | **rep_4** | **rep_5** | **average** | **sem^1^** |
| Breed_B | 27.47 | 26.10 | 27.11 | 27.34 | 27.59 | 27.12 | 0.27 |
| Breed_C | 28.02 | 28.52 | 27.14 | 28.28 | 28.50 | 28.09 | 0.25 |
| Breed_E | 27.05 | 27.55 | 28.76 | 27.06 | 26.82 | 27.45 | 0.35 |
| Comp_2 | 27.08 | 24.73 | 25.72 | 25.80 | 25.75 | 25.82 | 0.37 |
| Comp_3 | 28.26 | 26.64 | 24.63 | 26.78 | 27.69 | 26.80 | 0.62 |
| ^1^ Standard error mean. |  |  |  |  |  |  |  |
| ^2^ rep_1 to rep_5: repetitions. |  |  |  |  |  |  |  |

| Table 4. Residual variances for the pedigree-based model. | | | | | | | |
| --- | --- | --- | --- | --- | --- | --- | --- |
| **Population** | **rep_1** | **rep_2** | **rep_3** | **rep_4** | **rep_5** | **average** | **sem^1^** |
| Breed_B | 71.68 | 71.24 | 71.34 | 71.76 | 71.67 | 71.54 | 0.10 |
| Breed_C | 70.58 | 71.06 | 71.78 | 70.51 | 70.34 | 70.85 | 0.26 |
| Breed_E | 71.55 | 71.92 | 70.10 | 71.62 | 71.94 | 71.42 | 0.34 |
| Comp_2 | 72.28 | 73.80 | 73.05 | 72.94 | 73.30 | 73.07 | 0.25 |
| Comp_3 | 72.13 | 73.34 | 74.54 | 72.76 | 71.65 | 72.88 | 0.50 |
| ^1^ Standard error mean. |  |  |  |  |  |  |  |
| ^2^ rep_1 to rep_5: repetitions. |  |  |  |  |  |  |  |

| Table 5. Heritability for the pedigree-based model. | | | | | | | |
| --- | --- | --- | --- | --- | --- | --- | --- |
| **Population** | **rep_1** | **rep_2** | **rep_3** | **rep_4** | **rep_5** | **average** | **sem^1^** |
| Breed_B | 0.28 | 0.27 | 0.28 | 0.28 | 0.28 | 0.27 | 0.00 |
| Breed_C | 0.28 | 0.29 | 0.27 | 0.29 | 0.29 | 0.28 | 0.00 |
| Breed_E | 0.27 | 0.28 | 0.29 | 0.27 | 0.27 | 0.28 | 0.00 |
| Comp_2 | 0.27 | 0.25 | 0.26 | 0.26 | 0.26 | 0.26 | 0.00 |
| Comp_3 | 0.28 | 0.27 | 0.25 | 0.27 | 0.28 | 0.27 | 0.01 |
| ^1^ Standard error mean. |  |  |  |  |  |  |  |
| ^2^ rep_1 to rep_5: repetitions. |  |  |  |  |  |  |  |

Table 6. Variance components for all replicates under single-step GBLUP scenarios for the simulated population Breed_B.

| **Scenario^1^** | **Variance Component^2^** | **rep_1^4^** | **rep_2** | **rep_3** | **rep_4** | **rep_5** | **average** | **sem^3^** |
| --- | --- | --- | --- | --- | --- | --- | --- | --- |
| 600K | va | 27.18 | 26.04 | 30.16 | 30.17 | 24.62 | 27.63 | 1.11 |
|  | ve | 72.82 | 72.15 | 71.01 | 72.92 | 70.99 | 71.98 | 0.42 |
|  | h2 | 0.27 | 0.27 | 0.30 | 0.29 | 0.26 | 0.28 | 0.01 |
| 50K | va | 27.70 | 30.17 | 29.69 | 28.71 | 30.52 | 29.36 | 0.51 |
|  | ve | 72.29 | 69.29 | 72.33 | 69.22 | 72.31 | 71.09 | 0.75 |
|  | h2 | 0.28 | 0.30 | 0.29 | 0.29 | 0.30 | 0.29 | 0.00 |
| IPS_LD01 | va | 26.47 | 29.83 | 27.75 | 29.22 | 26.98 | 28.05 | 0.64 |
|  | ve | 73.53 | 69.52 | 70.27 | 75.69 | 72.62 | 72.33 | 1.12 |
|  | h2 | 0.26 | 0.30 | 0.28 | 0.28 | 0.27 | 0.28 | 0.01 |
| IPS_LD03 | va | 27.68 | 30.31 | 25.28 | 30.91 | 29.00 | 28.64 | 1.01 |
|  | ve | 72.32 | 69.22 | 72.72 | 69.35 | 73.75 | 71.47 | 0.92 |
|  | h2 | 0.28 | 0.30 | 0.26 | 0.31 | 0.28 | 0.29 | 0.01 |
| IPS_LD06 | va | 26.01 | 27.13 | 26.57 | 26.61 | 24.23 | 26.11 | 0.50 |
|  | ve | 73.99 | 72.60 | 74.46 | 75.51 | 75.09 | 74.33 | 0.51 |
|  | h2 | 0.26 | 0.27 | 0.26 | 0.26 | 0.24 | 0.26 | 0.00 |
| PS_LD01 | va | 25.08 | 30.05 | 25.33 | 30.98 | 27.57 | 27.80 | 1.20 |
|  | ve | 74.92 | 71.99 | 75.46 | 72.07 | 73.93 | 73.67 | 0.71 |
|  | h2 | 0.25 | 0.29 | 0.25 | 0.30 | 0.27 | 0.27 | 0.01 |
| PS_LD03 | va | 24.20 | 25.25 | 28.59 | 24.69 | 27.87 | 26.12 | 0.88 |
|  | ve | 75.80 | 75.42 | 74.28 | 75.90 | 74.82 | 75.24 | 0.31 |
|  | h2 | 0.24 | 0.25 | 0.28 | 0.25 | 0.27 | 0.26 | 0.01 |
| PS_LD06 | va | - | - | - | - | - | - | - |
|  | ve | - | - | - | - | - | - | - |
|  | h2 | - | - | - | - | - | - | - |
| IPS_2H_LD01 | va_1 | 17.85 | 15.82 | 19.48 | 16.72 | 17.54 | 17.48 | 0.61 |
|  | va_2 | 8.52 | 9.75 | 7.40 | 9.11 | 8.88 | 8.73 | 0.39 |
|  | ve | 72.79 | 71.99 | 72.09 | 73.13 | 72.78 | 72.56 | 0.22 |
|  | h2_1 | 0.18 | 0.16 | 0.20 | 0.17 | 0.18 | 0.18 | 0.01 |
|  | h2_2 | 0.09 | 0.10 | 0.07 | 0.09 | 0.09 | 0.09 | 0.00 |
|  | h2_1+2 | 0.27 | 0.26 | 0.27 | 0.26 | 0.27 | 0.27 | 0.00 |
| IPS_2H_LD03 | va_1 | 21.21 | 22.11 | 22.66 | 21.01 | 20.04 | 21.41 | 0.46 |
|  | va_2 | 5.06 | 3.03 | 4.18 | 4.68 | 6.41 | 4.67 | 0.55 |
|  | ve | 72.90 | 72.37 | 72.16 | 73.29 | 72.80 | 72.70 | 0.20 |
|  | h2_1 | 0.21 | 0.23 | 0.23 | 0.21 | 0.20 | 0.22 | 0.00 |
|  | h2_2 | 0.05 | 0.03 | 0.04 | 0.05 | 0.06 | 0.05 | 0.01 |
|  | h2_1+2 | 0.26 | 0.26 | 0.27 | 0.26 | 0.27 | 0.26 | 0.00 |
| IPS_2H_LD06 | va_1 | - | - | - | - | - | - | - |
|  | va_2 | - | - | - | - | - | - | - |
|  | ve | - | - | - | - | - | - | - |
|  | h2_1 | - | - | - | - | - | - | - |
|  | h2_2 | - | - | - | - | - | - | - |
|  | h2_1+2 | - | - | - | - | - | - | - |
| ^1^ 600K: high density panel; 50K: moderate panel; IPS_LD01, IPS_LD03 and IPS_LD06: independent and pseudo-SNPs from blocks with linkage disequilibrium (LD) threshold of 0.1, 0.3 and 0.6 in one relationship matrix, respectively; PS_LD01, PS_LD03 and PS_LD06: pseudo-SNPs from blocks with LD threshold of 0.1, 0.3 and 0.6, respectively; IPS_2H_LD01, IPS_2H_LD03 and IPS_2H_LD06: independent and pseudo-SNPs from blocks with LD threshold of 0.1, 0.3 and 0.6 in two relationship matrices, respectively. | | | | | | | | |
| ^2^ va: additive variance. ve: residual variance. h2: heritability. va_1: additive variance for the first component of the GEBV (non-clustered SNPs). va_2: additive variance for the second component of the GEBV (pseudo-SNPs). h2_1: heritability for the first component of the GEBV (non-clustered SNPs). h2_2: heritability for the second component of the GEBV (pseudo-SNPs). h2_1+2: heritability of the first plus second components of the GEBV (non-clustered + pseudo-SNPs). | | | | | | | | |
| ^3^ Standard error mean. | | | | | | | | |
| ^4^ rep_1 to rep_5: repetitions. | | | | | | | | |

Table 7. Variance components for all replicates under single-step GBLUP scenarios for the simulated population Breed_C.

| **Scenario^1^** | **Variance Component^2^** | **rep_1^4^** | **rep_2** | **rep_3** | **rep_4** | **rep_5** | **average** | **sem^3^** |
| --- | --- | --- | --- | --- | --- | --- | --- | --- |
| 600K | va | 28.51 | 25.39 | 25.68 | 25.58 | 26.17 | 26.26 | 0.57 |
|  | ve | 71.49 | 70.53 | 69.80 | 71.57 | 75.55 | 71.79 | 1.00 |
|  | h2 | 0.29 | 0.26 | 0.27 | 0.26 | 0.26 | 0.27 | 0.00 |
| 50K | va | 27.44 | 25.39 | 26.66 | 28.46 | 27.38 | 27.07 | 0.51 |
|  | ve | 72.56 | 74.77 | 69.47 | 69.73 | 73.85 | 72.08 | 1.07 |
|  | h2 | 0.27 | 0.25 | 0.28 | 0.29 | 0.27 | 0.27 | 0.01 |
| IPS_LD01 | va | 28.35 | 30.36 | 24.14 | 28.60 | 24.36 | 27.16 | 1.24 |
|  | ve | 71.65 | 71.39 | 75.72 | 72.47 | 69.59 | 72.16 | 1.01 |
|  | h2 | 0.28 | 0.30 | 0.24 | 0.28 | 0.26 | 0.27 | 0.01 |
| IPS_LD03 | va | 26.36 | 24.90 | 28.06 | 28.49 | 30.44 | 27.65 | 0.95 |
|  | ve | 73.64 | 75.85 | 70.84 | 72.09 | 70.36 | 72.56 | 1.00 |
|  | h2 | 0.26 | 0.25 | 0.28 | 0.28 | 0.30 | 0.28 | 0.01 |
| IPS_LD06 | va | - | - | - | - | - | - | - |
|  | ve | - | - | - | - | - | - | - |
|  | h2 | - | - | - | - | - | - | - |
| PS_LD01 | va | 26.72 | 30.78 | 27.62 | 28.09 | 27.46 | 28.13 | 0.70 |
|  | ve | 73.28 | 72.78 | 71.68 | 69.35 | 72.21 | 71.86 | 0.68 |
|  | h2 | 0.27 | 0.30 | 0.28 | 0.29 | 0.28 | 0.28 | 0.01 |
| PS_LD03 | va | 26.14 | 24.66 | 28.23 | 28.86 | 24.62 | 26.50 | 0.88 |
|  | ve | 73.86 | 75.23 | 75.92 | 69.89 | 72.63 | 73.51 | 1.07 |
|  | h2 | 0.26 | 0.25 | 0.27 | 0.29 | 0.25 | 0.26 | 0.01 |
| PS_LD06 | va | - | - | - | - | - | - | - |
|  | ve | - | - | - | - | - | - | - |
|  | h2 | - | - | - | - | - | - | - |
| IPS_2H_LD01 | va_1 | 17.30 | 17.68 | 14.53 | 13.99 | 18.26 | 16.35 | 0.87 |
|  | va_2 | 9.93 | 9.90 | 11.33 | 12.54 | 8.99 | 10.54 | 0.63 |
|  | ve | 71.21 | 71.54 | 72.56 | 71.76 | 71.25 | 71.66 | 0.25 |
|  | h2_1 | 0.18 | 0.18 | 0.15 | 0.14 | 0.19 | 0.17 | 0.01 |
|  | h2_2 | 0.10 | 0.10 | 0.12 | 0.13 | 0.09 | 0.11 | 0.01 |
|  | h2_1+2 | 0.28 | 0.28 | 0.26 | 0.27 | 0.28 | 0.27 | 0.00 |
| IPS_2H_LD03 | va_1 | - | - | - | - | - | - | - |
|  | va_2 | - | - | - | - | - | - | - |
|  | ve | - | - | - | - | - | - | - |
|  | h2_1 | - | - | - | - | - | - | - |
|  | h2_2 | - | - | - | - | - | - | - |
|  | h2_1+2 | - | - | - | - | - | - | - |
| IPS_2H_LD06 | va_1 | - | - | - | - | - | - | - |
|  | va_2 | - | - | - | - | - | - | - |
|  | ve | - | - | - | - | - | - | - |
|  | h2_1 | - | - | - | - | - | - | - |
|  | h2_2 | - | - | - | - | - | - | - |
|  | h2_1+2 | - | - | - | - | - | - | - |
| ^1^ 600K: high density panel; 50K: moderate panel; IPS_LD01, IPS_LD03 and IPS_LD06: independent and pseudo-SNPs from blocks with linkage disequilibrium (LD) threshold of 0.1, 0.3 and 0.6 in one relationship matrix, respectively; PS_LD01, PS_LD03 and PS_LD06: pseudo-SNPs from blocks with LD threshold of 0.1, 0.3 and 0.6, respectively; IPS_2H_LD01, IPS_2H_LD03 and IPS_2H_LD06: independent and pseudo-SNPs from blocks with LD threshold of 0.1, 0.3 and 0.6 in two relationship matrices, respectively. | | | | | | | | |
| ^2^ va: additive variance. ve: residual variance. h2: heritability. va_1: additive variance for the first component of the GEBV (non-clustered SNPs). va_2: additive variance for the second component of the GEBV (pseudo-SNPs). h2_1: heritability for the first component of the GEBV (non-clustered SNPs). h2_2: heritability for the second component of the GEBV (pseudo-SNPs). h2_1+2: heritability of the first plus second components of the GEBV (non-clustered + pseudo-SNPs). | | | | | | | | |
| ^3^ Standard error mean. | | | | | | | | |
| ^4^ rep_1 to rep_5: repetitions. | | | | | | | | |

Table 8. Variance components for all replicates under single-step GBLUP scenarios for the simulated population Breed_E.

| **Scenario^1^** | **Variance Component^2^** | **rep_1^4^** | **rep_2** | **rep_3** | **rep_4** | **rep_5** | **average** | **sem^3^** |
| --- | --- | --- | --- | --- | --- | --- | --- | --- |
| 600K | va | 26.89 | 30.37 | 25.56 | 27.09 | 25.03 | 26.99 | 0.93 |
|  | ve | 73.11 | 74.41 | 73.51 | 74.68 | 73.30 | 73.80 | 0.31 |
|  | h2 | 0.27 | 0.29 | 0.26 | 0.27 | 0.25 | 0.27 | 0.01 |
| 50K | va | 26.93 | 29.99 | 25.64 | 28.19 | 29.95 | 28.14 | 0.85 |
|  | ve | 73.07 | 72.60 | 75.85 | 70.48 | 70.32 | 72.46 | 1.01 |
|  | h2 | 0.27 | 0.29 | 0.25 | 0.29 | 0.30 | 0.28 | 0.01 |
| IPS_LD01 | va | 25.38 | 27.22 | 27.42 | 30.15 | 27.89 | 27.61 | 0.76 |
|  | ve | 74.62 | 73.25 | 73.41 | 74.49 | 75.57 | 74.27 | 0.43 |
|  | h2 | 0.25 | 0.27 | 0.27 | 0.29 | 0.27 | 0.27 | 0.01 |
| IPS_LD03 | va | 25.03 | 25.47 | 25.88 | 24.10 | 26.86 | 25.47 | 0.46 |
|  | ve | 74.97 | 74.94 | 69.15 | 69.71 | 73.29 | 72.41 | 1.26 |
|  | h2 | 0.25 | 0.25 | 0.27 | 0.26 | 0.27 | 0.26 | 0.00 |
| IPS_LD06 | va | - | - | - | - | - | - | - |
|  | ve | - | - | - | - | - | - | - |
|  | h2 | - | - | - | - | - | - | - |
| PS_LD01 | va | 25.50 | 30.05 | 24.79 | 26.91 | 26.68 | 26.78 | 0.90 |
|  | ve | 74.50 | 73.26 | 70.81 | 74.33 | 69.73 | 72.53 | 0.96 |
|  | h2 | 0.25 | 0.29 | 0.26 | 0.27 | 0.28 | 0.27 | 0.01 |
| PS_LD03 | va | 24.19 | 24.84 | 28.02 | 26.65 | 24.30 | 25.60 | 0.75 |
|  | ve | 75.81 | 72.40 | 72.63 | 69.09 | 75.74 | 73.13 | 1.25 |
|  | h2 | 0.24 | 0.26 | 0.28 | 0.28 | 0.24 | 0.26 | 0.01 |
| PS_LD06 | va | - | - | - | - | - | - | - |
|  | ve | - | - | - | - | - | - | - |
|  | h2 | - | - | - | - | - | - | - |
| IPS_2H_LD01 | va_1 | 17.62 | 15.28 | 19.34 | 16.88 | 17.04 | 17.23 | 0.65 |
|  | va_2 | 7.94 | 10.66 | 7.88 | 8.87 | 8.27 | 8.72 | 0.51 |
|  | ve | 72.63 | 72.83 | 71.17 | 72.52 | 73.04 | 72.44 | 0.33 |
|  | h2_1 | 0.18 | 0.15 | 0.20 | 0.17 | 0.17 | 0.18 | 0.01 |
|  | h2_2 | 0.81 | 0.11 | 0.08 | 0.09 | 0.08 | 0.23 | 0.14 |
|  | h2_1+2 | 0.26 | 0.26 | 0.28 | 0.26 | 0.26 | 0.26 | 0.00 |
| IPS_2H_LD03 | va_1 | 24.85 | 24.26 | 20.33 | 21.12 | 22.13 | 22.54 | 0.88 |
|  | va_2 | 0.59 | 1.41 | 0.01 | 1.20 | 1.70 | 0.98 | 0.30 |
|  | ve | 72.78 | 73.10 | 77.91 | 77.67 | 76.20 | 75.53 | 1.10 |
|  | h2_1 | 0.25 | 0.25 | 0.21 | 0.21 | 0.22 | 0.23 | 0.01 |
|  | h2_2 | 0.01 | 0.01 | 0.01 | 0.01 | 0.02 | 0.01 | 0.00 |
|  | h2_1+2 | 0.26 | 0.26 | 0.22 | 0.22 | 0.24 | 0.24 | 0.01 |
| IPS_2H_LD06 | va_1 | - | - | - | - | - | - | - |
|  | va_2 | - | - | - | - | - | - | - |
|  | ve | - | - | - | - | - | - | - |
|  | h2_1 | - | - | - | - | - | - | - |
|  | h2_2 | - | - | - | - | - | - | - |
|  | h2_1+2 | - | - | - | - | - | - | - |
| ^1^ 600K: high density panel; 50K: moderate panel; IPS_LD01, IPS_LD03 and IPS_LD06: independent and pseudo-SNPs from blocks with linkage disequilibrium (LD) threshold of 0.1, 0.3 and 0.6 in one relationship matrix, respectively; PS_LD01, PS_LD03 and PS_LD06: pseudo-SNPs from blocks with LD threshold of 0.1, 0.3 and 0.6, respectively; IPS_2H_LD01, IPS_2H_LD03 and IPS_2H_LD06: independent and pseudo-SNPs from blocks with LD threshold of 0.1, 0.3 and 0.6 in two relationship matrices, respectively. | | | | | | | | |
| ^2^ va: additive variance. ve: residual variance. h2: heritability. va_1: additive variance for the first component of the GEBV (non-clustered SNPs). va_2: additive variance for the second component of the GEBV (pseudo-SNPs). h2_1: heritability for the first component of the GEBV (non-clustered SNPs). h2_2: heritability for the second component of the GEBV (pseudo-SNPs). h2_1+2: heritability of the first plus second components of the GEBV (non-clustered + pseudo-SNPs). | | | | | | | | |
| ^3^ Standard error mean. | | | | | | | | |
| ^4^ rep_1 to rep_5: repetitions. | | | | | | | | |

Table 9. Variance components for all replicates under single-step GBLUP scenarios for the simulated population Comp_2.

| **Scenario^1^** | **Variance Component^2^** | **rep_1^4^** | **rep_2** | **rep_3** | **rep_4** | **rep_5** | **average** | **sem^3^** |
| --- | --- | --- | --- | --- | --- | --- | --- | --- |
| 600K | va | 27.77 | 30.70 | 25.16 | 27.06 | 28.42 | 27.82 | 0.90 |
|  | ve | 72.21 | 75.11 | 75.49 | 69.11 | 74.36 | 73.26 | 1.18 |
|  | h2 | 0.28 | 0.29 | 0.25 | 0.28 | 0.28 | 0.28 | 0.01 |
| 50K | va | 26.11 | 29.76 | 28.22 | 26.06 | 26.92 | 27.42 | 0.70 |
|  | ve | 73.89 | 74.92 | 73.01 | 70.49 | 69.83 | 72.43 | 0.98 |
|  | h2 | 0.26 | 0.28 | 0.28 | 0.27 | 0.28 | 0.27 | 0.00 |
| IPS_LD01 | va | 27.42 | 28.59 | 27.23 | 26.72 | 30.05 | 28.00 | 0.60 |
|  | ve | 72.58 | 69.67 | 70.32 | 71.95 | 74.94 | 71.89 | 0.93 |
|  | h2 | 0.27 | 0.29 | 0.28 | 0.27 | 0.29 | 0.28 | 0.00 |
| IPS_LD03 | va | 26.21 | 30.10 | 25.77 | 30.53 | 25.00 | 27.52 | 1.16 |
|  | ve | 73.79 | 75.11 | 73.92 | 71.31 | 72.65 | 73.36 | 0.64 |
|  | h2 | 0.26 | 0.29 | 0.26 | 0.30 | 0.26 | 0.27 | 0.01 |
| IPS_LD06 | va | - | - | - | - | - | - | - |
|  | ve | - | - | - | - | - | - | - |
|  | h2 | - | - | - | - | - | - | - |
| PS_LD01 | va | 25.38 | 29.45 | 25.07 | 25.67 | 25.59 | 26.23 | 0.81 |
|  | ve | 74.62 | 70.49 | 74.16 | 69.98 | 75.91 | 73.03 | 1.18 |
|  | h2 | 0.25 | 0.29 | 0.25 | 0.27 | 0.25 | 0.26 | 0.01 |
| PS_LD03 | va | 25.76 | 26.56 | 26.66 | 28.31 | 26.05 | 26.67 | 0.44 |
|  | ve | 74.24 | 70.65 | 73.13 | 74.52 | 71.63 | 72.83 | 0.75 |
|  | h2 | 0.26 | 0.27 | 0.27 | 0.28 | 0.27 | 0.27 | 0.00 |
| PS_LD06 | va | - | - | - | - | - | - | - |
|  | ve | - | - | - | - | - | - | - |
|  | h2 | - | - | - | - | - | - | - |
| IPS_2H_LD01 | va_1 | 17.16 | 14.19 | 15.64 | 16.72 | 16.81 | 16.10 | 0.54 |
|  | va_2 | 9.26 | 10.09 | 9.14 | 8.81 | 8.98 | 9.26 | 0.22 |
|  | ve | 72.98 | 74.31 | 73.94 | 73.42 | 73.43 | 73.62 | 0.23 |
|  | h2_1 | 0.17 | 0.14 | 0.16 | 0.17 | 0.17 | 0.16 | 0.01 |
|  | h2_2 | 0.09 | 0.10 | 0.09 | 0.09 | 0.09 | 0.09 | 0.00 |
|  | h2_1+2 | 0.27 | 0.25 | 0.25 | 0.26 | 0.26 | 0.26 | 0.00 |
| IPS_2H_LD03 | va_1 | - | - | - | - | - | - | - |
|  | va_2 | - | - | - | - | - | - | - |
|  | ve | - | - | - | - | - | - | - |
|  | h2_1 | - | - | - | - | - | - | - |
|  | h2_2 | - | - | - | - | - | - | - |
|  | h2_1+2 | - | - | - | - | - | - | - |
| IPS_2H_LD06 | va_1 | - | - | - | - | - | - | - |
|  | va_2 | - | - | - | - | - | - | - |
|  | ve | - | - | - | - | - | - | - |
|  | h2_1 | - | - | - | - | - | - | - |
|  | h2_2 | - | - | - | - | - | - | - |
|  | h2_1+2 | - | - | - | - | - | - | - |
| ^1^ 600K: high density panel; 50K: moderate panel; IPS_LD01, IPS_LD03 and IPS_LD06: independent and pseudo-SNPs from blocks with linkage disequilibrium (LD) threshold of 0.1, 0.3 and 0.6 in one relationship matrix, respectively; PS_LD01, PS_LD03 and PS_LD06: pseudo-SNPs from blocks with LD threshold of 0.1, 0.3 and 0.6, respectively; IPS_2H_LD01, IPS_2H_LD03 and IPS_2H_LD06: independent and pseudo-SNPs from blocks with LD threshold of 0.1, 0.3 and 0.6 in two relationship matrices, respectively. | | | | | | | | |
| ^2^ va: additive variance. ve: residual variance. h2: heritability. va_1: additive variance for the first component of the GEBV (non-clustered SNPs). va_2: additive variance for the second component of the GEBV (pseudo-SNPs). h2_1: heritability for the first component of the GEBV (non-clustered SNPs). h2_2: heritability for the second component of the GEBV (pseudo-SNPs). h2_1+2: heritability of the first plus second components of the GEBV (non-clustered + pseudo-SNPs). | | | | | | | | |
| ^3^ Standard error mean. | | | | | | | | |
| ^4^ rep_1 to rep_5: repetitions. | | | | | | | | |

Table 10. Variance components for all replicates under single-step GBLUP scenarios for the simulated population Comp_3.

| **Scenario^1^** | **Variance Component^2^** | **rep_1^4^** | **rep_2** | **rep_3** | **rep_4** | **rep_5** | **average** | **sem^3^** |
| --- | --- | --- | --- | --- | --- | --- | --- | --- |
| 600K | va | 29.64 | 30.64 | 30.32 | 27.52 | 26.93 | 29.01 | 0.75 |
|  | ve | 70.36 | 71.70 | 71.29 | 72.70 | 74.18 | 72.05 | 0.65 |
|  | h2 | 0.30 | 0.30 | 0.30 | 0.27 | 0.27 | 0.29 | 0.01 |
| 50K | va | 29.20 | 25.88 | 27.15 | 25.20 | 28.91 | 27.27 | 0.79 |
|  | ve | 70.80 | 72.69 | 72.07 | 74.68 | 72.92 | 72.63 | 0.63 |
|  | h2 | 0.29 | 0.26 | 0.27 | 0.25 | 0.28 | 0.27 | 0.01 |
| IPS_LD01 | va | 28.24 | 27.28 | 27.92 | 27.52 | 24.01 | 27.00 | 0.76 |
|  | ve | 71.76 | 69.69 | 70.47 | 71.52 | 74.94 | 71.68 | 0.90 |
|  | h2 | 0.28 | 0.28 | 0.28 | 0.28 | 0.24 | 0.27 | 0.01 |
| IPS_LD03 | va | 27.31 | 27.37 | 25.91 | 26.49 | 25.89 | 26.59 | 0.32 |
|  | ve | 72.69 | 75.16 | 72.02 | 71.76 | 74.46 | 73.22 | 0.68 |
|  | h2 | 0.27 | 0.27 | 0.26 | 0.27 | 0.26 | 0.27 | 0.00 |
| IPS_LD06 | va | - | - | - | - | - | - | - |
|  | ve | - | - | - | - | - | - | - |
|  | h2 | - | - | - | - | - | - | - |
| PS_LD01 | va | 26.66 | 26.24 | 28.74 | 30.13 | 27.87 | 27.93 | 0.71 |
|  | ve | 73.33 | 73.76 | 75.61 | 71.87 | 69.10 | 72.74 | 1.09 |
|  | h2 | 0.26 | 0.26 | 0.28 | 0.30 | 0.29 | 0.28 | 0.01 |
| PS_LD03 | va | 24.10 | 28.35 | 26.31 | 27.35 | 30.36 | 27.30 | 1.04 |
|  | ve | 75.90 | 74.27 | 70.11 | 74.73 | 72.67 | 73.54 | 1.00 |
|  | h2 | 0.24 | 0.28 | 0.27 | 0.27 | 0.29 | 0.27 | 0.01 |
| PS_LD06 | va | - | - | - | - | - | - | - |
|  | ve | - | - | - | - | - | - | - |
|  | h2 | - | - | - | - | - | - | - |
| IPS_2H_LD01 | va_1 | 14.73 | 16.85 | 13.75 | 14.44 | 16.59 | 15.27 | 0.61 |
|  | va_2 | 13.17 | 8.12 | 11.00 | 12.48 | 10.94 | 11.14 | 0.87 |
|  | ve | 72.61 | 74.43 | 74.85 | 72.73 | 71.93 | 73.31 | 0.56 |
|  | h2_1 | 0.15 | 0.17 | 0.14 | 0.14 | 0.17 | 0.15 | 0.01 |
|  | h2_2 | 0.13 | 0.08 | 0.11 | 0.13 | 0.11 | 0.11 | 0.01 |
|  | h2_1+2 | 0.28 | 0.25 | 0.25 | 0.27 | 0.28 | 0.26 | 0.01 |
| IPS_2H_LD03 | va_1 | - | - | - | - | - | - | - |
|  | va_2 | - | - | - | - | - | - | - |
|  | ve | - | - | - | - | - | - | - |
|  | h2_1 | - | - | - | - | - | - | - |
|  | h2_2 | - | - | - | - | - | - | - |
|  | h2_1+2 | - | - | - | - | - | - | - |
| IPS_2H_LD06 | va_1 | - | - | - | - | - | - | - |
|  | va_2 | - | - | - | - | - | - | - |
|  | ve | - | - | - | - | - | - | - |
|  | h2_1 | - | - | - | - | - | - | - |
|  | h2_2 | - | - | - | - | - | - | - |
|  | h2_1+2 | - | - | - | - | - | - | - |
| ^1^ 600K: high density panel; 50K: moderate panel; IPS_LD01, IPS_LD03 and IPS_LD06: independent and pseudo-SNPs from blocks with linkage disequilibrium (LD) threshold of 0.1, 0.3 and 0.6 in one relationship matrix, respectively; PS_LD01, PS_LD03 and PS_LD06: pseudo-SNPs from blocks with LD threshold of 0.1, 0.3 and 0.6, respectively; IPS_2H_LD01, IPS_2H_LD03 and IPS_2H_LD06: independent and pseudo-SNPs from blocks with LD threshold of 0.1, 0.3 and 0.6 in two relationship matrices, respectively. | | | | | | | | |
| ^2^ va: additive variance. ve: residual variance. h2: heritability. va_1: additive variance for the first component of the GEBV (non-clustered SNPs). va_2: additive variance for the second component of the GEBV (pseudo-SNPs). h2_1: heritability for the first component of the GEBV (non-clustered SNPs). h2_2: heritability for the second component of the GEBV (pseudo-SNPs). h2_1+2: heritability of the first plus second components of the GEBV (non-clustered + pseudo-SNPs). | | | | | | | | |
| ^3^ Standard error mean. | | | | | | | | |
| ^4^ rep_1 to rep_5: repetitions. | | | | | | | | |


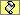

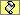

Supplement: Supplementary file 5 [file Table3.DOC]
