# Supplementary material for: A Comprehensive Comparison of Haplotype-Based Single-Step Genomic Predictions in Livestock Populations With Different Genetic Diversity Levels: A Simulation Study
Source: Front Genet. 2021 Oct 14;12:729867. doi: 10.3389/fgene.2021.729867 (PMC8551834; doi:10.3389/fgene.2021.729867)
Supplement: Supplementary file 7 [file Table2.DOCX]

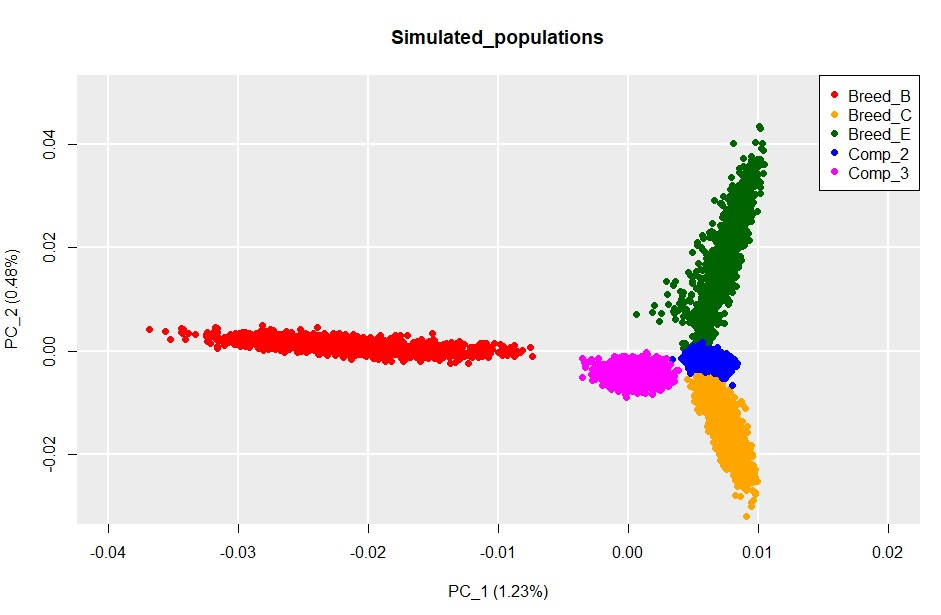


**Supplementary Figure 1.** First two principal components (PC) of the genomic relationship matrix created using Plink v.1.9 from genotyped individuals of the five simulated populations used to test the effect of haplotype predictions. Breed_B, Breed_C, Breed_E: simulated pure breeds; Comp_2: composite breed from two breeds; Comp_3: composite breed from three breeds.


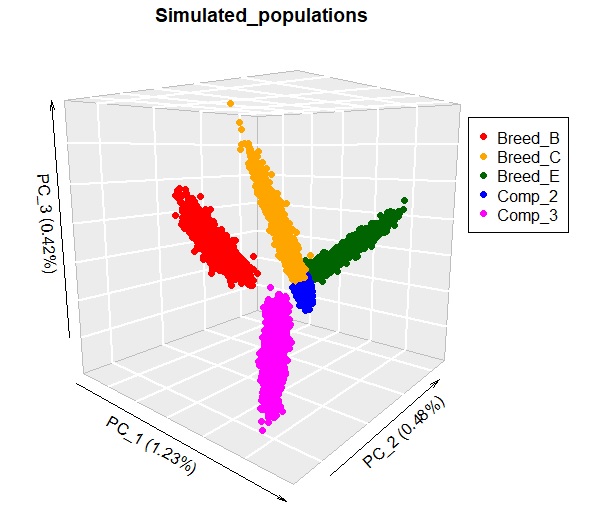


**Supplementary Figure 2.** First three principal components (PC) of the genomic relationship matrix created using Plink v.1.9 from genotyped individuals of the five simulated populations used to test the effect of haplotype predictions. Breed_B, Breed_C, Breed_E: simulated pure breeds; Comp_2: composite breed from two breeds; Comp_3: composite breed from three breeds.


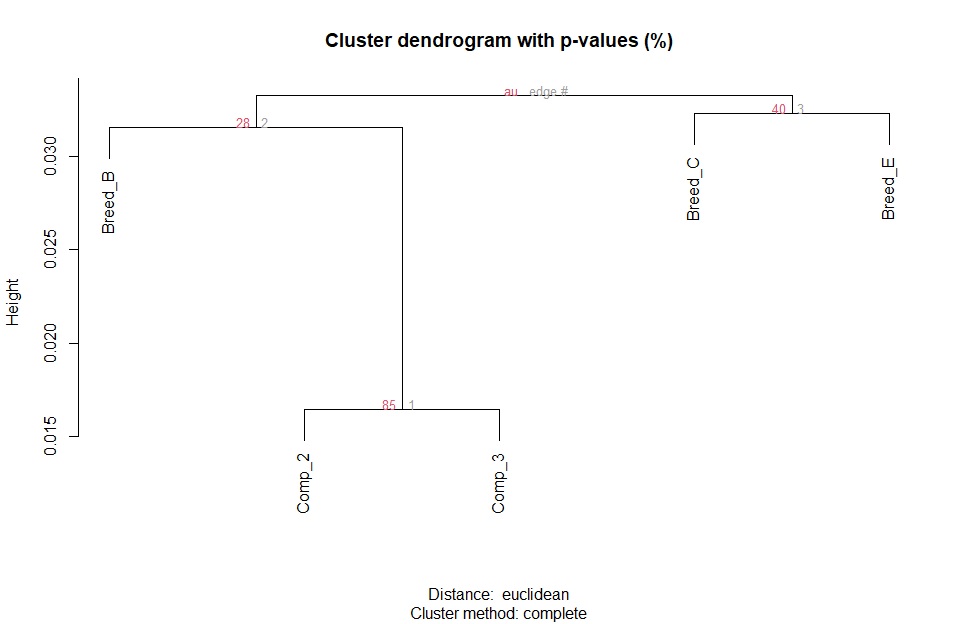


**Supplementary Figure 3.** Dendrogram of the five simulated populations using the first three principal components (PC) of the genomic relationship matrix created using Plink v.1.9 from genotyped individuals of the five simulated populations used to test the effect of haplotype predictions. Ten thousand bootstrap samples were used to compute the percentages of approximated unbiased test (au, red values). No clusters observed considering 95% as strong enough support a cluster. Breed_B, Breed_C, Breed_E: simulated pure breeds; Comp_2: composite breed from two breeds; Comp_3: composite breed from three breeds.
