# Supplementary material for: A Comprehensive Comparison of Haplotype-Based Single-Step Genomic Predictions in Livestock Populations With Different Genetic Diversity Levels: A Simulation Study
Source: Front Genet. 2021 Oct 14;12:729867. doi: 10.3389/fgene.2021.729867 (PMC8551834; doi:10.3389/fgene.2021.729867)
Supplement: Supplementary file 8 [file Table10.DOCX]

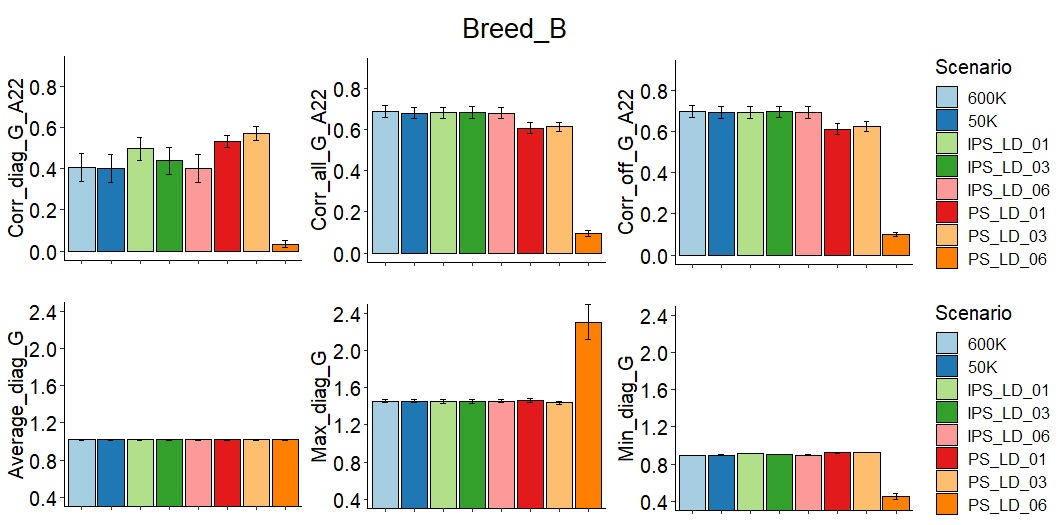
 Figure 1. Correlations between diagonal, all and off diagonal elements of the genomic (G) and pedigree (A22) relationship matrices (Corr_diag_G_A22, Corr_all_G_A22 and Corr_off_G_A22, respectively) and average, maximum and minimum values of G matrix diagonal elements (Average_diag_G, Max_diag_G and Min_diag_G) using SNPs or haplotypes. Breed_B: simulated pure breed with lower genetic diversity. 600K: high density panel; 50K: moderate panel; IPS_LD01, IPS_LD03 and IPS_LD06: independent and pseudo-SNPs from blocks with linkage disequilibrium (LD) threshold of 0.1, 0.3 and 0.6, respectively; PS_LD01, PS_LD03 and PS_LD06: pseudo-SNPs from blocks with LD threshold of 0.1, 0.3 and 0.6, respectively.


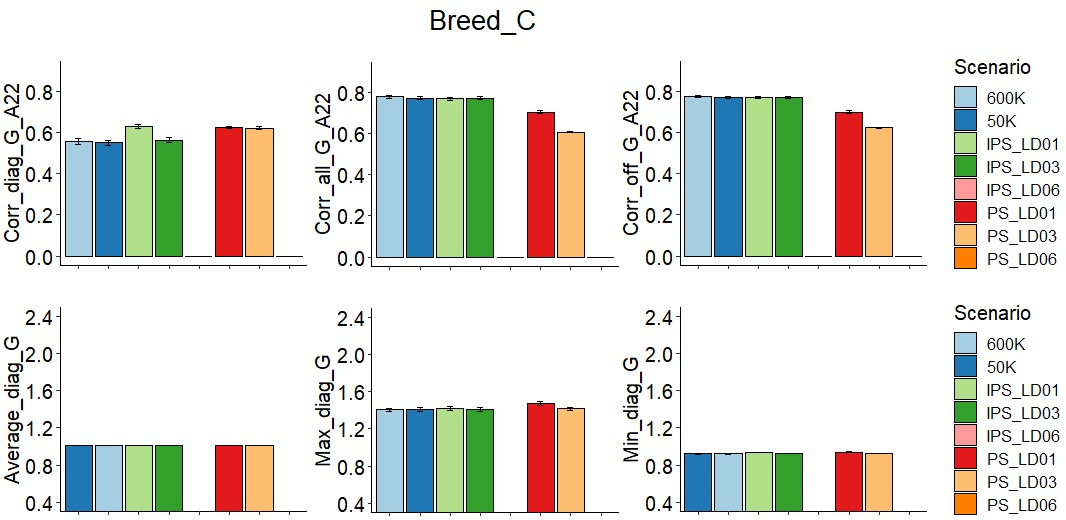
 Figure 2. Correlations between diagonal, all and off diagonal elements of the genomic (G) and pedigree (A22) relationship matrices (Corr_diag_G_A22, Corr_all_G_A22 and Corr_off_G_A22, respectively) and average, maximum and minimum values of G matrix diagonal elements (Average_diag_G, Max_diag_G and Min_diag_G) using SNPs or haplotypes. Breed_C: simulated pure breed with intermediary founder population and moderate genetic diversity. 600K: high density panel; 50K: moderate panel; IPS_LD01, IPS_LD03 and IPS_LD06: independent and pseudo-SNPs from blocks with linkage disequilibrium (LD) threshold of 0.1, 0.3 and 0.6, respectively; PS_LD01, PS_LD03 and PS_LD06: pseudo-SNPs from blocks with LD threshold of 0.1, 0.3 and 0.6, respectively. No results shown for the PS_LD06 because no pseudo-SNPs were obtained for this scenario.


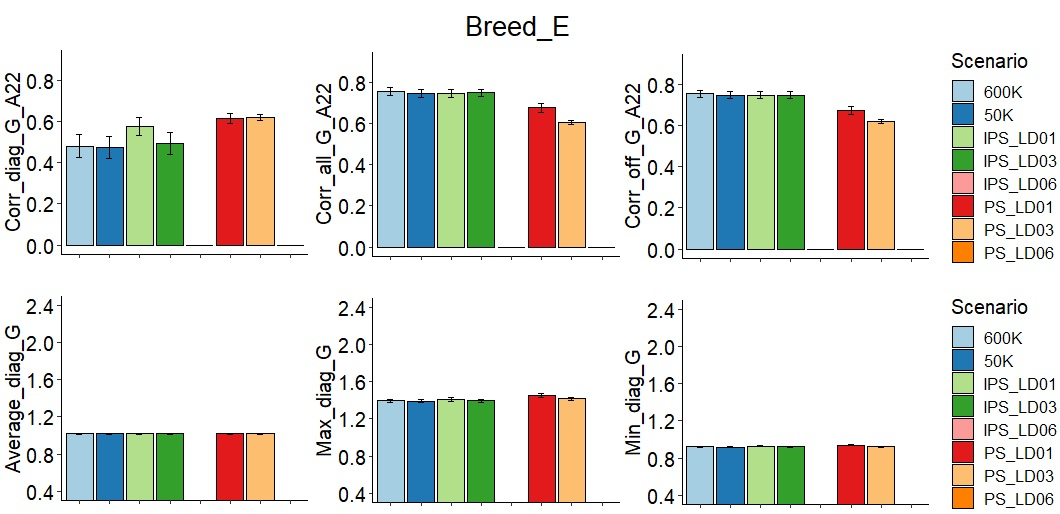
 Figure 3. Correlations between diagonal, all and off diagonal elements of the genomic (G) and pedigree (A22) relationship matrices (Corr_diag_G_A22, Corr_all_G_A22 and Corr_off_G_A22, respectively) and average, maximum and minimum values of G matrix diagonal elements (Average_diag_G, Max_diag_G and Min_diag_G) using SNPs or haplotypes. Breed_E: simulated pure breed with larger founder population and moderate genetic diversity. 600K: high density panel; 50K: moderate panel; IPS_LD01, IPS_LD03 and IPS_LD06: independent and pseudo-SNPs from blocks with linkage disequilibrium (LD) threshold of 0.1, 0.3 and 0.6, respectively; PS_LD01, PS_LD03 and PS_LD06: pseudo-SNPs from blocks with LD threshold of 0.1, 0.3 and 0.6, respectively. No results shown for the PS_LD06 because no pseudo-SNPs were obtained for this scenario.


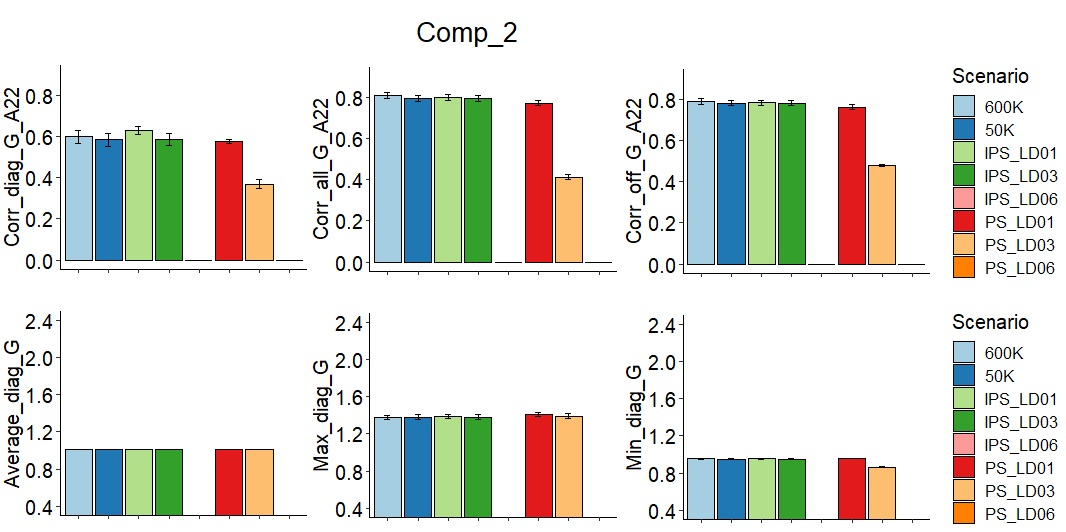
 Figure 4. Correlations between diagonal, all and off diagonal elements of the genomic (G) and pedigree (A22) relationship matrices (Corr_diag_G_A22, Corr_all_G_A22 and Corr_off_G_A22, respectively) and average, maximum and minimum values of G matrix diagonal elements (Average_diag_G, Max_diag_G and Min_diag_G) using SNPs or haplotypes. Comp_2: simulated composite breed from two breeds and high genetic diversity. 600K: high density panel; 50K: moderate panel; IPS_LD01, IPS_LD03 and IPS_LD06: independent and pseudo-SNPs from blocks with linkage disequilibrium (LD) threshold of 0.1, 0.3 and 0.6, respectively; PS_LD01, PS_LD03 and PS_LD06: pseudo-SNPs from blocks with LD threshold of 0.1, 0.3 and 0.6, respectively. No results shown for the PS_LD06 because no pseudo-SNPs were obtained for this scenario.


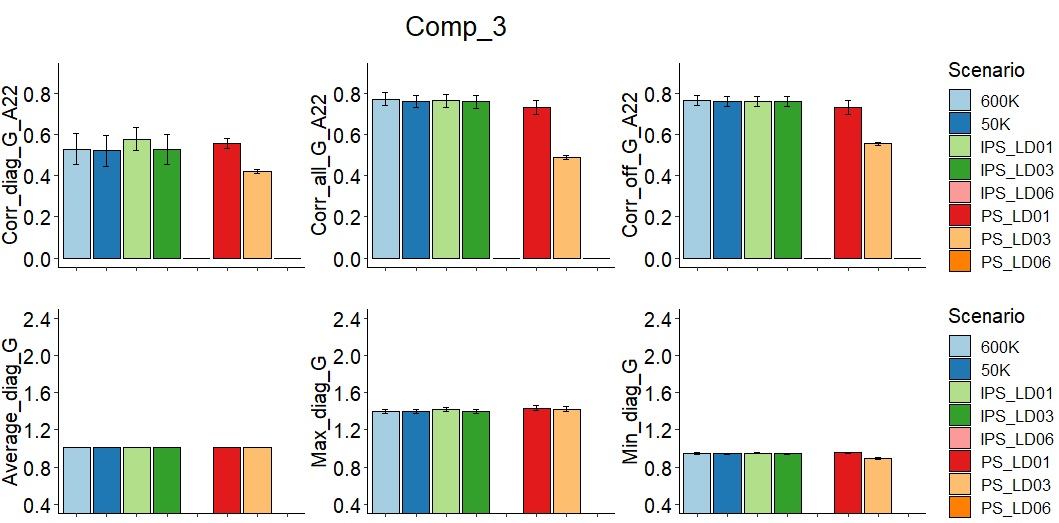
 Figure 5. Correlations between diagonal, all and off diagonal elements of the genomic (G) and pedigree (A22) relationship matrices (Corr_diag_G_A22, Corr_all_G_A22 and Corr_off_G_A22, respectively) and average, maximum and minimum values of G matrix diagonal elements (Average_diag_G, Max_diag_G and Min_diag_G) using SNPs or haplotypes. Comp_3: simulated composite breed from three breeds and high genetic diversity. 600K: high density panel; 50K: moderate panel; IPS_LD01, IPS_LD03 and IPS_LD06: independent and pseudo-SNPs from blocks with linkage disequilibrium (LD) threshold of 0.1, 0.3 and 0.6, respectively; PS_LD01, PS_LD03 and PS_LD06: pseudo-SNPs from blocks with LD threshold of 0.1, 0.3 and 0.6, respectively. No results shown for the PS_LD06 because no pseudo-SNPs were obtained for this scenario.
