# Supplementary material for: A Comprehensive Comparison of Haplotype-Based Single-Step Genomic Predictions in Livestock Populations With Different Genetic Diversity Levels: A Simulation Study
Source: Front Genet. 2021 Oct 14;12:729867. doi: 10.3389/fgene.2021.729867 (PMC8551834; doi:10.3389/fgene.2021.729867)

# Accuracies and bias of genomic predictions using SNPs or halotypes: low heritability

## [Accuracies and bias of genomic predictions using SNPs or halotypes: low heritability](#content)

- [Summary](#summary)

Andre Araujo

30/05/2021

# Summary

In this file is shown the values for each repetition and also the mean and standard error mean for the accuracies and bias of genomic prediction with SNPs or haplotypes in the simulated populations used on the research entitled A comprehensive comparison of single-step genomic BLUP approaches fitting SNPs or haplotypes in populations with different genetic diversity levels: a simulation study, by Araujo et al.

| Table 1. Prediction accuracy with SNPs or haplotypes in the Breed_B population. | | | | | | | |
| --- | --- | --- | --- | --- | --- | --- | --- |
| **Scenario** | **rep_1** | **rep_2** | **rep_3** | **rep_4** | **rep_5** | **average** | **sem^1^** |
| 600K | 0.35 | 0.30 | 0.30 | 0.37 | 0.28 | 0.32 | 0.02 |
| 50K | 0.36 | 0.30 | 0.29 | 0.36 | 0.29 | 0.32 | 0.02 |
| IPS_LD01 | 0.35 | 0.29 | 0.28 | 0.34 | 0.27 | 0.31 | 0.02 |
| IPS_LD03 | 0.36 | 0.29 | 0.28 | 0.35 | 0.27 | 0.31 | 0.02 |
| IPS_LD06 | 0.36 | 0.30 | 0.29 | 0.36 | 0.29 | 0.32 | 0.02 |
| PS_LD01 | 0.32 | 0.25 | 0.26 | 0.31 | 0.23 | 0.27 | 0.02 |
| PS_LD03 | 0.32 | 0.23 | 0.26 | 0.27 | 0.24 | 0.27 | 0.02 |
| PS_LD06 | 0.00 | 0.00 | 0.00 | 0.00 | 0.00 | 0.00 | 0.00 |
| IPS_2H_LD01 | 0.35 | 0.29 | 0.28 | 0.34 | 0.27 | 0.31 | 0.02 |
| IPS_2H_LD03 | 0.35 | 0.27 | 0.28 | 0.33 | 0.27 | 0.30 | 0.02 |
| IPS_2H_LD06 | 0.00 | 0.00 | 0.00 | 0.00 | 0.00 | 0.00 | 0.00 |
| ^1^ Standard error mean. |  |  |  |  |  |  |  |
| ^2^ rep_1 to rep_5: repetitions. |  |  |  |  |  |  |  |
| ^3^ Zero in all replicates, mean and sem means no reults shown. |  |  |  |  |  |  |  |
| ^4^ 600K: high density panel; 50K: moderate panel; IPS_LD01, IPS_LD03 and IPS_LD06: independent and pseudo-SNPs from blocks with linkage disequilibrium (LD) threshold of 0.1, 0.3 and 0.6 in one relationship matrix, respectively; PS_LD01, PS_LD03 and PS_LD06: pseudo-SNPs from blocks with LD threshold of 0.1, 0.3 and 0.6, respectively; IPS_2H_LD01, IPS_2H_LD03 and IPS_2H_LD06: independent and pseudo-SNPs from blocks with LD threshold of 0.1, 0.3 and 0.6 in two relationship matrices, respectively. |  |  |  |  |  |  |  |

| Table 2. Prediction accuracy with SNPs or haplotypes in the Breed_C population. | | | | | | | |
| --- | --- | --- | --- | --- | --- | --- | --- |
| **Scenario** | **rep_1** | **rep_2** | **rep_3** | **rep_4** | **rep_5** | **average** | **sem^1^** |
| 600K | 0.26 | 0.33 | 0.31 | 0.36 | 0.33 | 0.32 | 0.02 |
| 50K | 0.25 | 0.33 | 0.31 | 0.35 | 0.32 | 0.31 | 0.02 |
| IPS_LD01 | 0.22 | 0.32 | 0.28 | 0.36 | 0.30 | 0.30 | 0.02 |
| IPS_LD03 | 0.24 | 0.33 | 0.30 | 0.35 | 0.31 | 0.31 | 0.02 |
| IPS_LD06 | 0.00 | 0.00 | 0.00 | 0.00 | 0.00 | 0.00 | 0.00 |
| PS_LD01 | 0.21 | 0.30 | 0.24 | 0.33 | 0.27 | 0.27 | 0.02 |
| PS_LD03 | 0.14 | 0.21 | 0.17 | 0.27 | 0.24 | 0.20 | 0.02 |
| PS_LD06 | 0.00 | 0.00 | 0.00 | 0.00 | 0.00 | 0.00 | 0.00 |
| IPS_2H_LD01 | 0.23 | 0.32 | 0.28 | 0.35 | 0.30 | 0.30 | 0.02 |
| IPS_2H_LD03 | 0.23 | 0.32 | 0.29 | 0.35 | 0.31 | 0.30 | 0.02 |
| IPS_2H_LD06 | 0.00 | 0.00 | 0.00 | 0.00 | 0.00 | 0.00 | 0.00 |
| ^1^ Standard error mean. |  |  |  |  |  |  |  |
| ^2^ rep_1 to rep_5: repetitions. |  |  |  |  |  |  |  |
| ^3^ Zero in all replicates, mean and sem means no reults shown. |  |  |  |  |  |  |  |
| ^4^ 600K: high density panel; 50K: moderate panel; IPS_LD01, IPS_LD03 and IPS_LD06: independent and pseudo-SNPs from blocks with linkage disequilibrium (LD) threshold of 0.1, 0.3 and 0.6 in one relationship matrix, respectively; PS_LD01, PS_LD03 and PS_LD06: pseudo-SNPs from blocks with LD threshold of 0.1, 0.3 and 0.6, respectively; IPS_2H_LD01, IPS_2H_LD03 and IPS_2H_LD06: independent and pseudo-SNPs from blocks with LD threshold of 0.1, 0.3 and 0.6 in two relationship matrices, respectively. |  |  |  |  |  |  |  |

| Table 3. Prediction accuracy with SNPs or haplotypes in the Breed_E population. | | | | | | | |
| --- | --- | --- | --- | --- | --- | --- | --- |
| **Scenario** | **rep_1** | **rep_2** | **rep_3** | **rep_4** | **rep_5** | **average** | **sem^1^** |
| 600K | 0.33 | 0.30 | 0.32 | 0.25 | 0.30 | 0.30 | 0.01 |
| 50K | 0.32 | 0.30 | 0.31 | 0.25 | 0.29 | 0.29 | 0.01 |
| IPS_LD01 | 0.32 | 0.28 | 0.29 | 0.23 | 0.27 | 0.28 | 0.01 |
| IPS_LD03 | 0.32 | 0.29 | 0.30 | 0.24 | 0.29 | 0.29 | 0.01 |
| IPS_LD06 | 0.00 | 0.00 | 0.00 | 0.00 | 0.00 | 0.00 | 0.00 |
| PS_LD01 | 0.31 | 0.24 | 0.26 | 0.20 | 0.25 | 0.25 | 0.02 |
| PS_LD03 | 0.22 | 0.18 | 0.20 | 0.17 | 0.16 | 0.18 | 0.01 |
| PS_LD06 | 0.00 | 0.00 | 0.00 | 0.00 | 0.00 | 0.00 | 0.00 |
| IPS_2H_LD01 | 0.32 | 0.28 | 0.29 | 0.22 | 0.27 | 0.27 | 0.02 |
| IPS_2H_LD03 | 0.32 | 0.28 | 0.29 | 0.24 | 0.28 | 0.28 | 0.01 |
| IPS_2H_LD06 | 0.00 | 0.00 | 0.00 | 0.00 | 0.00 | 0.00 | 0.00 |
| ^1^ Standard error mean. |  |  |  |  |  |  |  |
| ^2^ rep_1 to rep_5: repetitions. |  |  |  |  |  |  |  |
| ^3^ Zero in all replicates, mean and sem means no reults shown. |  |  |  |  |  |  |  |
| ^4^ 600K: high density panel; 50K: moderate panel; IPS_LD01, IPS_LD03 and IPS_LD06: independent and pseudo-SNPs from blocks with linkage disequilibrium (LD) threshold of 0.1, 0.3 and 0.6 in one relationship matrix, respectively; PS_LD01, PS_LD03 and PS_LD06: pseudo-SNPs from blocks with LD threshold of 0.1, 0.3 and 0.6, respectively; IPS_2H_LD01, IPS_2H_LD03 and IPS_2H_LD06: independent and pseudo-SNPs from blocks with LD threshold of 0.1, 0.3 and 0.6 in two relationship matrices, respectively. |  |  |  |  |  |  |  |

| Table 4. Prediction accuracy with SNPs or haplotypes in the Comp_2 population. | | | | | | | |
| --- | --- | --- | --- | --- | --- | --- | --- |
| **Scenario** | **rep_1** | **rep_2** | **rep_3** | **rep_4** | **rep_5** | **average** | **sem^1^** |
| 600K | 0.21 | 0.29 | 0.25 | 0.26 | 0.23 | 0.25 | 0.01 |
| 50K | 0.20 | 0.28 | 0.24 | 0.24 | 0.23 | 0.24 | 0.01 |
| IPS_LD01 | 0.20 | 0.27 | 0.22 | 0.22 | 0.22 | 0.23 | 0.01 |
| IPS_LD03 | 0.20 | 0.28 | 0.24 | 0.24 | 0.23 | 0.24 | 0.01 |
| IPS_LD06 | 0.00 | 0.00 | 0.00 | 0.00 | 0.00 | 0.00 | 0.00 |
| PS_LD01 | 0.19 | 0.24 | 0.19 | 0.21 | 0.21 | 0.21 | 0.01 |
| PS_LD03 | 0.08 | 0.11 | 0.15 | 0.13 | 0.09 | 0.11 | 0.01 |
| PS_LD06 | 0.00 | 0.00 | 0.00 | 0.00 | 0.00 | 0.00 | 0.00 |
| IPS_2H_LD01 | 0.20 | 0.26 | 0.21 | 0.22 | 0.22 | 0.22 | 0.01 |
| IPS_2H_LD03 | 0.18 | 0.26 | 0.22 | 0.23 | 0.21 | 0.22 | 0.01 |
| IPS_2H_LD06 | 0.00 | 0.00 | 0.00 | 0.00 | 0.00 | 0.00 | 0.00 |
| ^1^ Standard error mean. |  |  |  |  |  |  |  |
| ^2^ rep_1 to rep_5: repetitions. |  |  |  |  |  |  |  |
| ^3^ Zero in all replicates, mean and sem means no reults shown. |  |  |  |  |  |  |  |
| ^4^ 600K: high density panel; 50K: moderate panel; IPS_LD01, IPS_LD03 and IPS_LD06: independent and pseudo-SNPs from blocks with linkage disequilibrium (LD) threshold of 0.1, 0.3 and 0.6 in one relationship matrix, respectively; PS_LD01, PS_LD03 and PS_LD06: pseudo-SNPs from blocks with LD threshold of 0.1, 0.3 and 0.6, respectively; IPS_2H_LD01, IPS_2H_LD03 and IPS_2H_LD06: independent and pseudo-SNPs from blocks with LD threshold of 0.1, 0.3 and 0.6 in two relationship matrices, respectively. |  |  |  |  |  |  |  |

| Table 5. Prediction accuracy with SNPs or haplotypes in the Comp_3 population. | | | | | | | |
| --- | --- | --- | --- | --- | --- | --- | --- |
| **Scenario** | **rep_1** | **rep_2** | **rep_3** | **rep_4** | **rep_5** | **average** | **sem^1^** |
| 600K | 0.21 | 0.27 | 0.25 | 0.26 | 0.27 | 0.25 | 0.01 |
| 50K | 0.20 | 0.26 | 0.24 | 0.26 | 0.26 | 0.24 | 0.01 |
| IPS_LD01 | 0.19 | 0.26 | 0.23 | 0.25 | 0.25 | 0.24 | 0.01 |
| IPS_LD03 | 0.20 | 0.26 | 0.24 | 0.26 | 0.26 | 0.24 | 0.01 |
| IPS_LD06 | 0.00 | 0.00 | 0.00 | 0.00 | 0.00 | 0.00 | 0.00 |
| PS_LD01 | 0.17 | 0.22 | 0.22 | 0.24 | 0.23 | 0.22 | 0.01 |
| PS_LD03 | 0.12 | 0.14 | 0.10 | 0.11 | 0.11 | 0.12 | 0.01 |
| PS_LD06 | 0.00 | 0.00 | 0.00 | 0.00 | 0.00 | 0.00 | 0.00 |
| IPS_2H_LD01 | 0.18 | 0.24 | 0.23 | 0.25 | 0.24 | 0.23 | 0.01 |
| IPS_2H_LD03 | 0.19 | 0.25 | 0.22 | 0.24 | 0.24 | 0.23 | 0.01 |
| IPS_2H_LD06 | 0.00 | 0.00 | 0.00 | 0.00 | 0.00 | 0.00 | 0.00 |
| ^1^ Standard error mean. |  |  |  |  |  |  |  |
| ^2^ rep_1 to rep_5: repetitions. |  |  |  |  |  |  |  |
| ^3^ Zero in all replicates, mean and sem means no reults shown. |  |  |  |  |  |  |  |
| ^4^ 600K: high density panel; 50K: moderate panel; IPS_LD01, IPS_LD03 and IPS_LD06: independent and pseudo-SNPs from blocks with linkage disequilibrium (LD) threshold of 0.1, 0.3 and 0.6 in one relationship matrix, respectively; PS_LD01, PS_LD03 and PS_LD06: pseudo-SNPs from blocks with LD threshold of 0.1, 0.3 and 0.6, respectively; IPS_2H_LD01, IPS_2H_LD03 and IPS_2H_LD06: independent and pseudo-SNPs from blocks with LD threshold of 0.1, 0.3 and 0.6 in two relationship matrices, respectively. |  |  |  |  |  |  |  |

| Table 6. Prediction bias with SNPs or haplotypes in the Breed_B population. | | | | | | | |
| --- | --- | --- | --- | --- | --- | --- | --- |
| **Scenario** | **rep_1** | **rep_2** | **rep_3** | **rep_4** | **rep_5** | **average** | **sem^1^** |
| 600K | -0.28 | -0.34 | -0.39 | -0.14 | -0.42 | -0.32 | 0.05 |
| 50K | -0.27 | -0.35 | -0.42 | -0.16 | -0.42 | -0.33 | 0.05 |
| IPS_LD01 | -0.26 | -0.37 | -0.41 | -0.21 | -0.45 | -0.34 | 0.04 |
| IPS_LD03 | -0.27 | -0.37 | -0.42 | -0.19 | -0.45 | -0.34 | 0.05 |
| IPS_LD06 | -0.27 | -0.35 | -0.42 | -0.16 | -0.42 | -0.33 | 0.05 |
| PS_LD01 | -0.30 | -0.46 | -0.47 | -0.29 | -0.52 | -0.41 | 0.05 |
| PS_LD03 | -0.34 | -0.51 | -0.45 | -0.40 | -0.50 | -0.44 | 0.03 |
| PS_LD06 | 0.00 | 0.00 | 0.00 | 0.00 | 0.00 | 0.00 | 0.00 |
| IPS_2H_LD01 | -0.28 | -0.42 | -0.42 | -0.25 | -0.44 | -0.36 | 0.04 |
| IPS_2H_LD03 | -0.28 | -0.45 | -0.43 | -0.28 | -0.45 | -0.38 | 0.04 |
| IPS_2H_LD06 | 0.00 | 0.00 | 0.00 | 0.00 | 0.00 | 0.00 | 0.00 |
| ^1^ Standard error mean. |  |  |  |  |  |  |  |
| ^2^ rep_1 to rep_5: repetitions. |  |  |  |  |  |  |  |
| ^3^ Zero in all replicates, mean and sem means no reults shown. |  |  |  |  |  |  |  |
| ^4^ 600K: high density panel; 50K: moderate panel; IPS_LD01, IPS_LD03 and IPS_LD06: independent and pseudo-SNPs from blocks with linkage disequilibrium (LD) threshold of 0.1, 0.3 and 0.6 in one relationship matrix, respectively; PS_LD01, PS_LD03 and PS_LD06: pseudo-SNPs from blocks with LD threshold of 0.1, 0.3 and 0.6, respectively; IPS_2H_LD01, IPS_2H_LD03 and IPS_2H_LD06: independent and pseudo-SNPs from blocks with LD threshold of 0.1, 0.3 and 0.6 in two relationship matrices, respectively. |  |  |  |  |  |  |  |

| Table 7. Prediction bias with SNPs or haplotypes in the Breed_C population. | | | | | | | |
| --- | --- | --- | --- | --- | --- | --- | --- |
| **Scenario** | **rep_1** | **rep_2** | **rep_3** | **rep_4** | **rep_5** | **average** | **sem^1^** |
| 600K | -0.34 | -0.19 | -0.31 | -0.14 | -0.33 | -0.26 | 0.04 |
| 50K | -0.36 | -0.20 | -0.33 | -0.19 | -0.34 | -0.28 | 0.04 |
| IPS_LD01 | -0.44 | -0.20 | -0.37 | -0.14 | -0.38 | -0.30 | 0.06 |
| IPS_LD03 | -0.39 | -0.19 | -0.34 | -0.17 | -0.36 | -0.29 | 0.04 |
| IPS_LD06 | 0.00 | 0.00 | 0.00 | 0.00 | 0.00 | 0.00 | 0.00 |
| PS_LD01 | -0.49 | -0.29 | -0.48 | -0.18 | -0.45 | -0.38 | 0.06 |
| PS_LD03 | -0.73 | -0.58 | -0.67 | -0.47 | -0.57 | -0.60 | 0.05 |
| PS_LD06 | 0.00 | 0.00 | 0.00 | 0.00 | 0.00 | 0.00 | 0.00 |
| IPS_2H_LD01 | -0.44 | -0.23 | -0.38 | -0.18 | -0.36 | -0.32 | 0.05 |
| IPS_2H_LD03 | -0.44 | -0.25 | -0.38 | -0.21 | -0.35 | -0.32 | 0.04 |
| IPS_2H_LD06 | 0.00 | 0.00 | 0.00 | 0.00 | 0.00 | 0.00 | 0.00 |
| ^1^ Standard error mean. |  |  |  |  |  |  |  |
| ^2^ rep_1 to rep_5: repetitions. |  |  |  |  |  |  |  |
| ^3^ Zero in all replicates, mean and sem means no reults shown. |  |  |  |  |  |  |  |
| ^4^ 600K: high density panel; 50K: moderate panel; IPS_LD01, IPS_LD03 and IPS_LD06: independent and pseudo-SNPs from blocks with linkage disequilibrium (LD) threshold of 0.1, 0.3 and 0.6 in one relationship matrix, respectively; PS_LD01, PS_LD03 and PS_LD06: pseudo-SNPs from blocks with LD threshold of 0.1, 0.3 and 0.6, respectively; IPS_2H_LD01, IPS_2H_LD03 and IPS_2H_LD06: independent and pseudo-SNPs from blocks with LD threshold of 0.1, 0.3 and 0.6 in two relationship matrices, respectively. |  |  |  |  |  |  |  |

| Table 8. Prediction bias with SNPs or haplotypes in the Breed_E population. | | | | | | | |
| --- | --- | --- | --- | --- | --- | --- | --- |
| **Scenario** | **rep_1** | **rep_2** | **rep_3** | **rep_4** | **rep_5** | **average** | **sem^1^** |
| 600K | -0.26 | -0.25 | -0.27 | -0.41 | -0.27 | -0.29 | 0.03 |
| 50K | -0.29 | -0.27 | -0.29 | -0.41 | -0.30 | -0.31 | 0.02 |
| IPS_LD01 | -0.28 | -0.29 | -0.31 | -0.44 | -0.33 | -0.33 | 0.03 |
| IPS_LD03 | -0.29 | -0.27 | -0.31 | -0.42 | -0.31 | -0.32 | 0.03 |
| IPS_LD06 | 0.00 | 0.00 | 0.00 | 0.00 | 0.00 | 0.00 | 0.00 |
| PS_LD01 | -0.31 | -0.40 | -0.38 | -0.53 | -0.42 | -0.41 | 0.04 |
| PS_LD03 | -0.59 | -0.61 | -0.60 | -0.64 | -0.67 | -0.62 | 0.01 |
| PS_LD06 | 0.00 | 0.00 | 0.00 | 0.00 | 0.00 | 0.00 | 0.00 |
| IPS_2H_LD01 | -0.31 | -0.37 | -0.35 | -0.50 | -0.38 | -0.38 | 0.03 |
| IPS_2H_LD03 | -0.32 | -0.35 | -0.35 | -0.45 | -0.36 | -0.37 | 0.02 |
| IPS_2H_LD06 | 0.00 | 0.00 | 0.00 | 0.00 | 0.00 | 0.00 | 0.00 |
| ^1^ Standard error mean. |  |  |  |  |  |  |  |
| ^2^ rep_1 to rep_5: repetitions. |  |  |  |  |  |  |  |
| ^3^ Zero in all replicates, mean and sem means no reults shown. |  |  |  |  |  |  |  |
| ^4^ 600K: high density panel; 50K: moderate panel; IPS_LD01, IPS_LD03 and IPS_LD06: independent and pseudo-SNPs from blocks with linkage disequilibrium (LD) threshold of 0.1, 0.3 and 0.6 in one relationship matrix, respectively; PS_LD01, PS_LD03 and PS_LD06: pseudo-SNPs from blocks with LD threshold of 0.1, 0.3 and 0.6, respectively; IPS_2H_LD01, IPS_2H_LD03 and IPS_2H_LD06: independent and pseudo-SNPs from blocks with LD threshold of 0.1, 0.3 and 0.6 in two relationship matrices, respectively. |  |  |  |  |  |  |  |

| Table 9. Prediction bias with SNPs or haplotypes in the Comp_2 population. | | | | | | | |
| --- | --- | --- | --- | --- | --- | --- | --- |
| **Scenario** | **rep_1** | **rep_2** | **rep_3** | **rep_4** | **rep_5** | **average** | **sem^1^** |
| 600K | -0.39 | -0.17 | -0.37 | -0.30 | -0.33 | -0.31 | 0.04 |
| 50K | -0.42 | -0.22 | -0.42 | -0.37 | -0.37 | -0.36 | 0.04 |
| IPS_LD01 | -0.43 | -0.24 | -0.46 | -0.41 | -0.38 | -0.38 | 0.04 |
| IPS_LD03 | -0.43 | -0.22 | -0.42 | -0.37 | -0.36 | -0.36 | 0.04 |
| IPS_LD06 | 0.00 | 0.00 | 0.00 | 0.00 | 0.00 | 0.00 | 0.00 |
| PS_LD01 | -0.46 | -0.30 | -0.52 | -0.46 | -0.42 | -0.43 | 0.04 |
| PS_LD03 | -0.83 | -0.76 | -0.71 | -0.75 | -0.82 | -0.78 | 0.02 |
| PS_LD06 | 0.00 | 0.00 | 0.00 | 0.00 | 0.00 | 0.00 | 0.00 |
| IPS_2H_LD01 | -0.44 | -0.34 | -0.49 | -0.44 | -0.41 | -0.43 | 0.03 |
| IPS_2H_LD03 | -0.48 | -0.35 | -0.46 | -0.42 | -0.47 | -0.44 | 0.02 |
| IPS_2H_LD06 | 0.00 | 0.00 | 0.00 | 0.00 | 0.00 | 0.00 | 0.00 |
| ^1^ Standard error mean. |  |  |  |  |  |  |  |
| ^2^ rep_1 to rep_5: repetitions. |  |  |  |  |  |  |  |
| ^3^ Zero in all replicates, mean and sem means no reults shown. |  |  |  |  |  |  |  |
| ^4^ 600K: high density panel; 50K: moderate panel; IPS_LD01, IPS_LD03 and IPS_LD06: independent and pseudo-SNPs from blocks with linkage disequilibrium (LD) threshold of 0.1, 0.3 and 0.6 in one relationship matrix, respectively; PS_LD01, PS_LD03 and PS_LD06: pseudo-SNPs from blocks with LD threshold of 0.1, 0.3 and 0.6, respectively; IPS_2H_LD01, IPS_2H_LD03 and IPS_2H_LD06: independent and pseudo-SNPs from blocks with LD threshold of 0.1, 0.3 and 0.6 in two relationship matrices, respectively. |  |  |  |  |  |  |  |

| Table 10. Prediction bias with SNPs or haplotypes in the Comp_3 population. | | | | | | | |
| --- | --- | --- | --- | --- | --- | --- | --- |
| **Scenario** | **rep_1** | **rep_2** | **rep_3** | **rep_4** | **rep_5** | **average** | **sem^1^** |
| 600K | -0.41 | -0.24 | -0.23 | -0.30 | -0.20 | -0.28 | 0.04 |
| 50K | -0.43 | -0.26 | -0.27 | -0.30 | -0.24 | -0.30 | 0.03 |
| IPS_LD01 | -0.45 | -0.26 | -0.27 | -0.31 | -0.24 | -0.30 | 0.04 |
| IPS_LD03 | -0.43 | -0.25 | -0.27 | -0.31 | -0.23 | -0.30 | 0.04 |
| IPS_LD06 | 0.00 | 0.00 | 0.00 | 0.00 | 0.00 | 0.00 | 0.00 |
| PS_LD01 | -0.49 | -0.39 | -0.28 | -0.34 | -0.27 | -0.35 | 0.04 |
| PS_LD03 | -0.75 | -0.67 | -0.77 | -0.77 | -0.76 | -0.75 | 0.02 |
| PS_LD06 | 0.00 | 0.00 | 0.00 | 0.00 | 0.00 | 0.00 | 0.00 |
| IPS_2H_LD01 | -0.49 | -0.38 | -0.35 | -0.33 | -0.27 | -0.36 | 0.04 |
| IPS_2H_LD03 | -0.48 | -0.32 | -0.40 | -0.37 | -0.31 | -0.37 | 0.03 |
| IPS_2H_LD06 | 0.00 | 0.00 | 0.00 | 0.00 | 0.00 | 0.00 | 0.00 |
| ^1^ Standard error mean. |  |  |  |  |  |  |  |
| ^2^ rep_1 to rep_5: repetitions. |  |  |  |  |  |  |  |
| ^3^ Zero in all replicates, mean and sem means no reults shown. |  |  |  |  |  |  |  |
| ^4^ 600K: high density panel; 50K: moderate panel; IPS_LD01, IPS_LD03 and IPS_LD06: independent and pseudo-SNPs from blocks with linkage disequilibrium (LD) threshold of 0.1, 0.3 and 0.6 in one relationship matrix, respectively; PS_LD01, PS_LD03 and PS_LD06: pseudo-SNPs from blocks with LD threshold of 0.1, 0.3 and 0.6, respectively; IPS_2H_LD01, IPS_2H_LD03 and IPS_2H_LD06: independent and pseudo-SNPs from blocks with LD threshold of 0.1, 0.3 and 0.6 in two relationship matrices, respectively. |  |  |  |  |  |  |  |


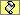

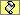

Supplement: Supplementary file 9 [file Table9.DOC]
